# A ParDE toxin–antitoxin system is responsible for the maintenance of the *Yersinia* virulence plasmid but not for type III secretion-associated growth inhibition

**DOI:** 10.3389/fcimb.2023.1166077

**Published:** 2023-05-09

**Authors:** Saskia Schott, Robina Scheuer, Francesca Ermoli, Timo Glatter, Elena Evguenieva-Hackenberg, Andreas Diepold

**Affiliations:** ^1^ Department of Ecophysiology, Max Planck Institute for Terrestrial Microbiology, Marburg, Germany; ^2^ Department of Microbiology and Molecular Biology, Justus Liebig University Gießen, Gießen, Germany; ^3^ Core Facility for Mass spectrometry & Proteomics, Max Planck Institute for Terrestrial Microbiology, Marburg, Germany

**Keywords:** bacterial secretion system, protein transport across membranes, pathogen-host cell interaction, *Yersinia enterocolitica*, toxin-antitoxin (TA) system, bacterial cell biology, bacterial virulence

## Abstract

Many Gram-negative pathogens utilize the type III secretion system (T3SS) to translocate virulence-promoting effector proteins into eukaryotic host cells. The activity of this system results in a severe reduction of bacterial growth and division, summarized as secretion-associated growth inhibition (SAGI). In *Yersinia enterocolitica*, the T3SS and related proteins are encoded on a virulence plasmid. We identified a ParDE-like toxin–antitoxin system on this virulence plasmid in genetic proximity to *yopE*, encoding a T3SS effector. Effectors are strongly upregulated upon activation of the T3SS, indicating a potential role of the ParDE system in the SAGI or maintenance of the virulence plasmid. Expression of the toxin ParE *in trans* resulted in reduced growth and elongated bacteria, highly reminiscent of the SAGI. Nevertheless, the activity of ParDE is not causal for the SAGI. T3SS activation did not influence ParDE activity; conversely, ParDE had no impact on T3SS assembly or activity itself. However, we found that ParDE ensures the presence of the T3SS across bacterial populations by reducing the loss of the virulence plasmid, especially under conditions relevant to infection. Despite this effect, a subset of bacteria lost the virulence plasmid and regained the ability to divide under secreting conditions, facilitating the possible emergence of T3SS-negative bacteria in late acute and persistent infections.

## Introduction

To interact with their environment and other organisms, bacteria evolved a variety of protein secretion mechanisms (Costa et al., 2015; Galán and Waksman, 2018; Denise et al., 2020). Many Gram-negative pathogens use the type III secretion system (T3SS)^1^ to deliver effector proteins into the host cell cytoplasm to manipulate the host immune response, resulting in increased bacterial survival chances (Büttner, 2012; Burkinshaw and Strynadka, 2014; Wagner et al., 2018). The T3SS is a multi-megadalton apparatus assembled from more than 20 proteins that span the bacterial inner, outer, and host cell membrane (Kubori et al., 1998; Burkinshaw and Strynadka, 2014) (**Supplementary Figure 1**). The responsible genes are usually encoded on genetic islands located on the chromosome or on virulence plasmids, such as the plasmid for Yersinia virulence (pYV) in *Yersinia*, and are distributed via horizontal gene transfer (Galán and Bliska, 1996; Abby and Rocha, 2012). In *Y. enterocolitica*, the expression of the T3SS is induced by the transcriptional regulator VirF^2^ upon a temperature shift to 37°C, the host body temperature (Mulder et al., 1989; Nuss et al., 2016; Schwiesow et al., 2016). The assembly process includes the secretion-dependent assembly of the membrane rings and export apparatus, followed by the formation of the extracellular needle and tip, whose subunits are exported by the T3SS itself (reviewed in Diepold and Wagner, 2014), which also requires its dynamic cytosolic complex (**Supplementary Figure 1**). Once the T3SS is fully assembled, it remains in a steady state until its activation is triggered (Enninga and Rosenshine, 2009). *In vivo*, secretion is induced upon sensing the host cell (Rosqvist et al., 1994; Cornelis et al., 1998). *In vitro*, this trigger can be mimicked by different chemicals and growth conditions, including calcium depletion in the extracellular environment (Michiels et al., 1990; Deng et al., 2005; Kim et al., 2005). At this point, protein secretion is initiated and the expression of effectors is massively upregulated (Enninga et al., 2005; Schlumberger et al., 2005; Wiley et al., 2007).

While almost all *Y. enterocolitica* (>98%) are T3SS-positive at 37°C (Wiley et al., 2007; Milne-Davies et al., 2019), only a subpopulation of *Salmonella enterica* and *Pseudomonas aeruginosa* expresses the T3SS during infection (Rietsch & Mekalanos, 2006; Sturm et al., 2011; Rundell et al., 2016). A possible reason is the phenomenon of secretion-associated growth inhibition (SAGI). Across organisms, T3SS-mediated protein secretion leads to a significant decrease or even a complete stop of bacterial cell growth and division (Carter et al., 1980; Sasakawa et al., 1986; Sturm et al., 2011). In fact, the SAGI, specifically the loss of growth of virulent *Yersinia* strains at 37°C under conditions now known to stimulate T3SS-dependent protein secretion^3^, was the observation that led to the discovery of the T3SS (Higuchi and Carlin, 1958; Kupferberg and Higuchi, 1958; Ben-Gurion and Shafferman, 1981; Ferber and Brubaker, 1981; Straley and Brubaker, 1981; Heesemann et al., 1984; Perry et al., 1986; Michiels et al., 1990) (reviewed by (Schneewind, 2016)). Although this effect is widely observed in unrelated bacteria, it is still unclear whether the SAGI is a passive consequence of secretion or an active mechanism.

In any case, the SAGI represents a high fitness cost, and since the utilization of the T3SS is indispensable for the virulence of *Yersinia*, as it is for many other pathogens (Coburn et al., 2007), the presence of the virulence plasmid must be actively safeguarded. One mechanism to ensure the stable maintenance of plasmids is the post-segregational killing of plasmid-free daughter cells mediated by toxin–antitoxin (TA) systems (Ogura and Hiraga, 1983). TA systems are widely distributed in bacteria and archaea and are classified into different types (Yamaguchi et al., 2011). Most known TA systems belong to the type II family (Karlowicz et al., 2016), which consists of two protein components: a stable toxin and an unstable antitoxin. Both encoding genes are organized in an operon and co-transcribed. The toxin is inactive in a complex with the antitoxin (Leplae et al., 2011). If the TA-encoding plasmid is not passed on to the daughter cell during cell division, the inherited antitoxin is quickly proteolytically degraded by Lon or Clp proteases (Williams and Hergenrother, 2012; Dubiel et al., 2018). Unbound toxins can then target essential bacterial processes such as DNA replication, protein synthesis, cell wall biosynthesis, and ATP synthesis (Unoson and Wagner, 2008; Yamaguchi et al., 2011). Depending on the mode of action of the respective toxin, loss of TA-containing plasmids can lead to reduced growth or even cell death (Van Melderen et al, 1994; Lehnherr and Yarmolinsky, 1995). In *Shigella flexneri*, the virulence plasmid pINV, which encodes the T3SS, is stabilized by three partially complementary TA systems (McVicker and Tang, 2017; Pilla et al., 2017; Martyn et al., 2022). No such mechanism is currently known for the *Yersinia* virulence plasmid.

Bioinformatic analyses identified a potential TA system on the virulence plasmid pYV of *Y. enterocolitica*. The protein pair displays homology to the toxin ParE and the antitoxin ParD, a type II family TA system (Roberts et al., 1994), and we will refer to them as ParE and ParD in this manuscript. ParE and ParD of different species have a low similarity at the amino acid level but share a common secondary structure (Ames et al., 2019). Free ParE inhibits gyrase activity (Roberts et al., 1994; Jiang et al., 2002; Fiebig et al., 2010; Kamruzzaman and Iredell, 2019) and, consequently, DNA replication and transcription.

The ParDE-encoding genes are located in close genetic proximity to the gene of a T3SS effector, *yopE*. Expression of effectors, and specifically *yopE*, is strongly upregulated upon the activation of secretion (Wiley et al., 2007). We, therefore, speculated that transcriptional interference between *yopE* and *parDE* or downstream effects of the ParE toxin might be the basis for the link between T3SS activation and lack of growth. In this study, we investigated the role of the ParDE TA system on bacterial growth and division, T3SS assembly and activity, and plasmid maintenance in *Y. enterocolitica*. Our results show that while both ParE expression and T3SS-dependent protein secretion similarly reduce growth and division, ParE is not a main factor in the SAGI. ParDE does not directly affect T3SS assembly and function but promotes retention of the virulence plasmid and consequently the presence of this crucial virulence factor despite its burden on the bacteria.

## Results

### Bioinformatic and transcriptional analysis of the T3SS-associated ParDE toxin–antitoxin system

**Figure 1:**
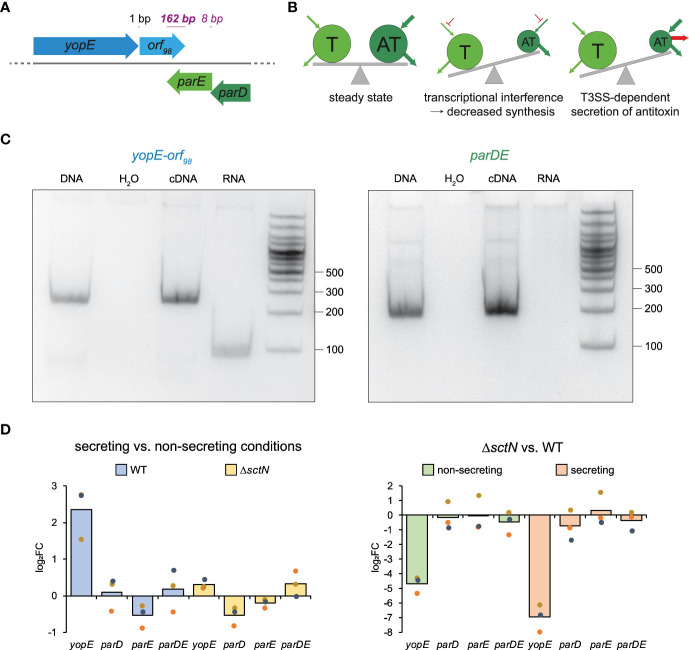
Genetic and possible functional relations between the ParDE TA system and the T3SS effector YopE. **(A)** Gene arrangement of *yopE*, *orf_98_*, *parE*, and *parD* on the *Y. enterocolitica* pYVe227 virulence plasmid (see **Supplementary Figure 2** for other *Yersinia* strains). Distances (black, regular font) and overlaps (purple, italic font) between adjacent genes are indicated. **(B)** Possible T3SS-associated effects on toxin–antitoxin levels that might cause secretion-associated growth inhibition (middle and right panels). Pools of toxin (T) and antitoxin (AT) are indicated by circles; arrows indicate biosynthesis and degradation of pools; see main text for details. **(C)** RT-PCR of *yopE-orf_98_* and *parDE*. Left panel, a forward primer located in *yopE* and a reverse primer located in *orf_98_* were used. Right panel, a forward primer located in *parD* and a reverse primer located in *parE* were used. The PCR template input is indicated at the top. The size standard (in nucleotides) is indicated on the right. Shown is a representative result of three independent experiments. **(D)** qRT-PCR analysis of changes (log_2_ fold changes (log_2_FC)) in the levels of the indicated transcripts in strains and conditions as indicated. The qRT-PCR reactions were performed in technical duplicates from three independent cultures.

We noticed that the virulence plasmid of *Y. enterocolitica* contains two adjacent genes with homology and a common predicted secondary structure to known ParDE TA systems. The genes are located downstream of the gene for the T3SS effector YopE on the opposite strand. Although the genes are not consistently annotated, possibly due to their small size (99 amino acids for ParE, 80 for ParD), we found them and their genetic environment to be highly conserved within *Yersinia* species: The *yopE* gene is directly followed by a small open reading frame of 98 amino acids (*orf_98_*, one nucleotide after *yopE*, **Figure 1A**) with no homologies outside *Yersinia* (*E* < 1 using a BLAST search (Altschul et al., 1990) on the nonredundant protein sequence database). On the opposite strand, *parD* and *parE* are encoded adjacently (eight nucleotides overlap). Intriguingly, *orf_98_* and *parE* on the two strands significantly overlap (162 bp, **Figure 1A**). This arrangement is conserved between different strains of *Y. enterocolitica*, *Y. pseudotuberculosis*, and *Y. pestis* (**Supplementary Figure 2**); only one analyzed strain differed by the insertion of a transposase-like element between *yopE* and *orf_98_*. This gene arrangement indicates the possibility of a direct link between ParDE and the SAGI (**Figure 1B**), either (i) by transcriptional or translational interference—where the massive increase of *yopE* transcription upon induction of secretion might inhibit the expression of *parDE* from the opposite strand—or (ii) by T3SS-dependent secretion of the antitoxin ParD. In both cases, ParE would be activated upon the initiation of secretion, which might cause SAGI.

To test if *yopE* and *orf_98_* are expressed in an operon, we performed a reverse transcription polymerase chain reaction and found that both genes are indeed co-transcribed, as was the case for *parD* and *parE*, which we used as a positive control (**Figure 1C**). As expected, *yopE* mRNA levels were strongly increased in wild-type strains (but not in strains lacking the T3SS ATPase SctN) under secreting conditions. In contrast, *parDE* mRNA levels were stable between secreting and non-secreting conditions and between wild-type and Δ*sctN* (**Figure 1D**).

### Characterization of the secretion-associated growth inhibition in *Yersinia enterocolitica*

Secretion through the T3SS is induced by host cell contact (Blocker et al., 2008), but in *Yersinia*, it can also be triggered by chelating Ca^2+^ in the growth medium, which possibly mimics the low calcium concentration in eukaryotic target cells (Kupferberg and Higuchi, 1958; Deng et al., 2005; Kim et al., 2005). Activation of the T3SS and secretion of effectors severely restricts growth in many species (Higuchi and Carlin, 1958; Carter et al., 1980; Sturm et al., 2011). We characterized this effect in *Y. enterocolitica* (**Supplementary Figure 3A**). The doubling time of secreting cells was increased by a factor of 2.7 (180.9 ± 19.6 min compared to 69.8 ± 5.5 min in nonsecreting bacteria, *p* < 0.001), and the maximum bacterial density (optical density at 600 nm (OD_600_)) was reduced by a factor of 4.8 (0.58 ± 0.05 vs. 2.85 ± 0.02, *p* < 0.001). Moreover, secretion resulted in elongated cell morphology (**Supplementary Figures 3B, C**). After 6 h of secretion, secreting bacteria were almost twice as long as nonsecreting bacteria (**Supplementary Figure 3C**). To exclude a secretion-independent effect of the medium, we performed growth tests for both a T3SS-defective strain (lacking the essential T3SS component SctD) and a strain secreting irrespective of cell contact and Ca^2+^ levels (which lacks the T3SS gatekeeper component SctW/YopN). Bacterial growth in these strains clearly correlated (inversely) with secretion activity rather than the medium (**Supplementary Figures 4A, B**). To distinguish if the presence of a functional T3SS under secreting conditions or protein secretion itself is responsible for the SAGI, we monitored the growth of a strain expressing a C-terminal EGFP fusion of a long synthetic effector protein based on the *Y. enterocolitica* effector YopO. Effectors are inserted into the needle in an unfolded state from their N-terminus (Akeda and Galán, 2005), and stably folded C-terminal domains such as EGFP can jam the injectisome and block secretion of other substrates (Feldman et al., 2002; Lee and Schneewind, 2002; Radics et al., 2014; LeBlanc et al., 2021). Upon expression of the jamming substrate, secretion was blocked despite functional T3SS and secretion-inducing conditions, which allowed the bacteria to grow similarly to bacteria under non-secreting conditions (**Supplementary Figure 4C**). Taken together, these results strongly support the hypothesis that T3SS-dependent secretion itself or a factor directly linked to it, rather than the energy expense of building injectisomes or factors in the medium, restricts bacterial growth.

### ParE expression mimics but does not cause secretion-associated growth inhibition

The close genetic proximity of *parDE* and the effector gene *yopE* prompted us to investigate if the ParDE TA system plays a role in the SAGI. To achieve this aim, we first compared the phenotypes of ParE expression and SAGI in terms of bacterial growth and cell morphology.

**Figure 2:**
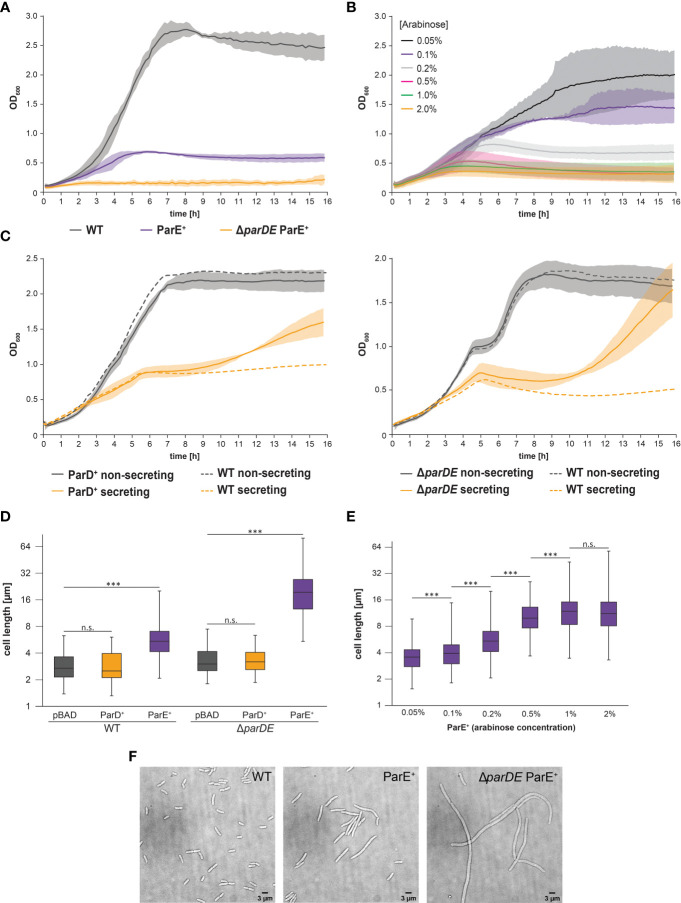
Expression of the toxin ParE limits growth and division but is not the basis of the secretion-associated growth inhibition. **(A)** Development of optical density at 600 nm (OD_600_) over time in the indicated strain backgrounds at 37°C under nonsecreting conditions. Compared to the wild-type bacteria (WT, gray), ParE expression (ParE^+^, purple) leads to growth inhibition, which is increased in the antitoxin-free Δ*parDE* background (orange). Likewise, the doubling time was extended (WT 69.8 ± 6.3 min; ParE^+^ 93.5 ± 3.7 min, p < 0.005; Δ*parDE* ParE^+^ 122.7 ± 19.7 min, p < 0.001). Average (solid lines) and standard deviation (shaded area) of three independent biological replicates, measured in technical quadruplicates. **(B)** Expression of ParE at 37°C under nonsecreting conditions results in growth inhibition in a concentration-dependent manner. Different ParE concentrations were induced by a varying amount of arabinose, as indicated. **(C)** Development of OD_600_ over time in the indicated strain backgrounds at 37°C. Average (solid lines) and standard deviation (shaded area) of three independent biological replicates, measured in technical quadruplicates with wild-type strains serving as controls (WT, dashed lines). Expression of antitoxin ParD (ParD^+^) (left) and absence of ParDE (Δ*parDE*) (right) do not significantly alter growth behavior within the first six hours after induction of T3SS expression by temperature shift to 37°C but lead to an increased OD_600_ under secreting conditions after this time (see main text for details). **(D)** Influence of ParDE on cell length at 37°C under nonsecreting conditions. Expression of ParE (purple) but not ParD (orange) led to elongated bacteria compared to the vector control (pBAD, gray). In antitoxin ParD-free cells (right), the effect was even more pronounced. Cell length values of 100 cells per biological replicate (n = 3) are shown as box plots on a logarithmic scale. The box extends from the lower to the upper quartile. The median is indicated by a vertical line, whiskers indicate minimal and maximal values. ^***^p < 0.001 in a two-tailed homoscedastic t-test. n.s., difference not statistically significant. **(E)** Expression of ParE at 37°C under nonsecreting conditions results in cell elongation in a concentration-dependent manner. Different ParE concentrations were induced by a varying amount of arabinose, as indicated. Cell length values of 100 cells per biological replicate (n = 3) are shown as box plots on a logarithmic scale. The box extends from the lower to the upper quartile. The median is indicated by a vertical line, whiskers indicate minimal and maximal values. ^***^p < 0.001 in a two-tailed homoscedastic t-test. n.s., difference not statistically significant. **(F)** Effect of additional ParE expression on cell morphology in wild-type and antitoxin-free cells (Δ*parDE*). Left, vector control. Representative images, n = 3.

In line with the function of ParD as an antitoxin, we were not able to generate a single *parD* mutant, while we readily obtained mutants in *parE* and *parDE* and expression plasmids for both proteins. ParD and ParE were expressed from a plasmid using an arabinose-inducible pBAD promoter. Expression of the toxin ParE resulted in increased doubling time (93.5 ± 3.6 min vs. 69.8 ± 5.5 min, *p* < 0.005) and a strongly decreased maximum culture density (0.70 ± 0.02 vs. 2.84 ± 0.09, *p* < 0.001) (**Figure 2A**). This effect was even more pronounced in a *parDE* deletion background, where the doubling time increased to 122.7 ± 17.3 min (*p* < 0.01 vs. wild-type) and the culture density only reached 0.24 ± 0.08 (*p* < 0.001), both reminiscent of the SAGI (**Supplementary Figure 3**). The effect of ParE on cell growth was concentration-dependent (**Figure 2B**); expression of ParD had no significant effect on growth (**Figure 2C**; **Supplementary Figure 5**).

Expression of the toxin ParE also led to an elongated cell morphology in *Y. enterocolitica* (**Figures 2D–F**), again reminiscent of the SAGI but more pronounced. Added expression of ParE in a wild-type strain background doubled the average cell length; antitoxin-free bacteria (Δ*parDE*) additionally expressing ParE were more than six times longer than wild-type cells (**Figures 2D–F**). Expression of ParD had no significant effect on cell morphology (**Figure 2D**; **Supplementary Figure 5**).

To test if ParE directly causes the SAGI, we challenged two consequences of this assumption: If the growth inhibition of secreting cells is caused by changes in the ratio of ParE and ParD (as would be the case in both scenarios depicted in **Figure 1B**), (i) additional expression of ParD should neutralize the free ParE toxin and enable secreting bacteria to grow like non-secreting ones, and (ii) creating a ParE-free strain by deleting the *parDE* operon should prevent the SAGI. Contrary to these assumptions, we found that expression of additional ParD from plasmid did not restore growth under secreting conditions within the first 6 h after induction of secretion (**Figure 2C**, left). In line with this finding, the SAGI still occurred in strains deleted for *parDE* (**Figure 2C**, right).

Notably, secreting bacteria lacking ParDE or overexpressing the antitoxin ParD resume growth and division after approximately six hours (**Figure 2C**), a possible indication for loss of the virulence plasmid under these conditions, which will be discussed later.

### The intracellular ParD/E expression ratio remains stable under secreting conditions

To test whether T3SS-dependent secretion of ParD changes the intracellular ParDE ratio, which could in turn decrease growth and division in secreting bacteria (**Figure 1B**, right), we analyzed the secretome (proteins in the culture supernatant) of secreting Y. enterocolitica by label-free mass spectrometry. Indeed, we detected the secretion of the antitoxin ParD. However, compared to *bona fide* T3SS cargo proteins such as effectors, ParD was only secreted to a low degree (**Supplementary Table 1**). To test if ParD secretion affects the cytosolic ratio of ParD and ParE, we next compared the proteome of secreting and nonsecreting bacteria by shotgun proteomics and label-free quantitative (LFQ) mass spectrometry. The relative amount of both proteins was not significantly affected during secretion (**Supplementary Table 2**). The limited secretion of ParD, therefore, does not affect the ParD/E ratio and is not causal for the SAGI. Likewise, the absence or overexpression of the main transcriptional activator of the *Y. enterocolitica* T3SS, VirF, did not affect the levels of ParD or ParE, in contrast to the levels of T3SS components (**Supplementary Table 3**).

### The T3SS-associated TA system does not directly influence T3SS assembly or activity

We next wanted to know if ParDE has a direct influence on T3SS assembly and activity in general. Using a functional translational EGFP-SctQ fusion expressed in its native genetic environment, we visualized the assembly of the T3SS under steady state (nonsecreting) and activated (secreting) conditions at 37°C. SctQ is one of the cytosolic components of the T3SS and localizes in foci at the bacterial surface, which represent assembled injectisomes (Diepold et al., 2010). The influence of ParDE was tested in bacteria lacking the whole *parDE* operon (Δ*parDE*) or the toxin *parE* (Δ*parE*), as well as in wild-type cells additionally expressing ParD (ParD^+^) or ParE (ParE^+^) from an arabinose-inducible vector. In none of the tested cases, fluorescence microscopy showed a significant impact of ParDE levels on the EGFP-SctQ pattern and thus assembly of the injectisome (**Supplementary Figure 6A**).

A possible influence of ParDE on T3SS *activity* was investigated by secretion and infection assays. Secretion assays monitor the secretion of the effector proteins into the medium, triggered by low calcium concentrations in the medium. Neither absence nor additional expression of ParD or ParE altered the amount and pattern of secreted effectors (**Supplementary Figure 6B**). Infection assays monitor the translocation of effectors into eukaryotic cells. The amount of translocation was measured by luciferase complementation mediated through translocation of the small part of the NanoLuc luciferase (HiBiT) fused to the T3SS secretion signal YopE_1–53_ into eukaryotic host cells (HeLa cells expressing the complementary large NanoLuc fragment (LgBiT)) (Lindner et al., 2020; Westerhausen et al., 2020). In the absence of ParDE or ParE alone, *Y. enterocolitica* was able to translocate the tagged T3SS effector into eukaryotic cells as efficiently as the wild-type control strain (**Supplementary Figure 6C**). Taken together, the ParDE system does not directly influence protein secretion or translocation by the T3SS.

### The ParDE system ensures virulence plasmid maintenance in *Y. enterocolitica*

**Figure 3:**
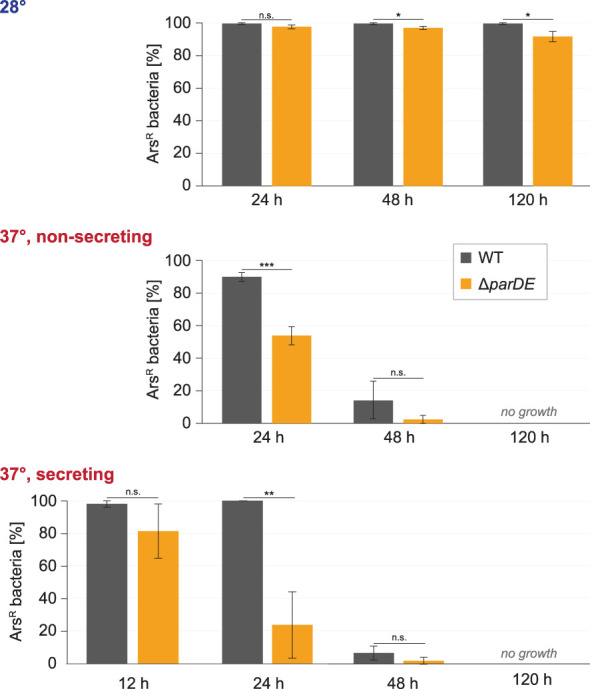
ParDE maintains the presence of the virulence plasmid. Retention of the virulence plasmid at the indicated conditions and time points was assayed by resistance against arsenate, which is encoded on the virulence plasmid. The presence of ParDE increased the plasmid’s stability under all tested conditions. *n* = 100 cells each in biological triplicates; error bars display standard variation. ^*^ *p* < 0.05; ^**^ *p* < 0.01; ^***^ *p* < 0.001 in a two-tailed homoscedastic *t*-test. n.s., difference not statistically significant.

**Figure 4:**
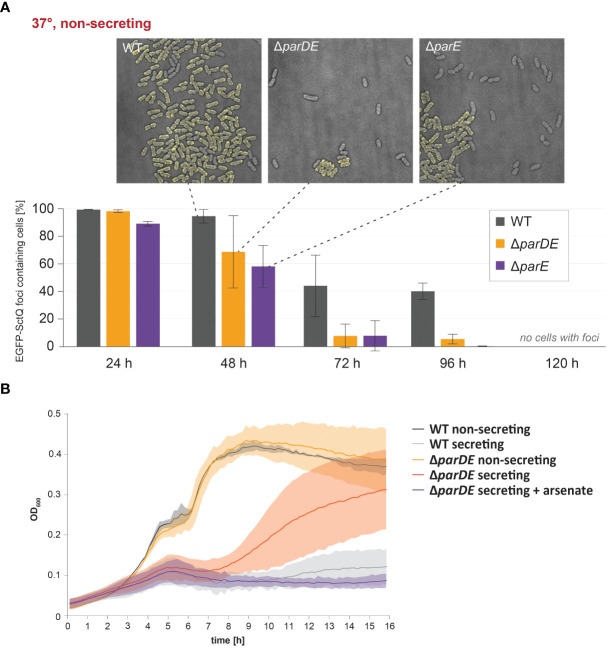
ParDE maintenance of the virulence plasmid regulates the assembly of the injectisome; in the absence of the system, fast-growing subpopulations emerge within 16 h. **(A)** Retention of the virulence plasmid at 37°C under nonsecreting conditions was tested by fluorescence microscopy. EGFP-SctQ is expressed from the virulence plasmid and visible as membrane-located foci. The presence of ParDE and ParE increased the plasmid stability. *n* = 100 cells each in biological triplicates; error bars display standard variation. Representative images (top) after 48 h under nonsecreting conditions. **(B)** At 37°C under secreting conditions, SAGI is released in cells missing the *parDE* operon (red), indicating a loss of virulence plasmid. The presence of arsenate in the medium (purple) selects for cells with virulence plasmids and prevents this effect (purple).

We next tested whether the ParDE system contributes to the maintenance of the virulence plasmid under different conditions. The presence of the virulence plasmid can be verified by selection on arsenate-containing solid media, as genes on the pYV plasmid encode for arsenate resistance mediating proteins (Iriarte and Cornelis, 1998). As it is conceivable that the loss of the virulence plasmid is correlated to its fitness costs, we performed these experiments (i) at 28°C, where expression of T3SS components is repressed (Michiels et al., 1990); (ii) at 37°C under nonsecreting conditions (presence of Ca^2+^ in medium), where the injectisome is assembled but no effector secretion takes place; and (iii) at 37°C under secreting conditions, where secretion is induced and the expression of injectisome components (Kudryashev et al., 2015) and especially effectors (Pettersson et al., 1996) is increased (**Figure 3**). The maintenance of the virulence plasmid was markedly influenced by these external conditions. At 28°C, where the virulence plasmid was retained over a long time in wild-type *Y. enterocolitica* (99.7% pYV^+^ after 5 days), the absence of the ParDE TA system led to higher rates of plasmid loss (91.7% pYV^+^ after 5 days) (**Figure 3**, top). A more significant loss was observed at 37°C at nonsecreting conditions (**Figure 3**, center) and secreting conditions (**Figure 3**, bottom). After 5 days, all tested bacteria had lost the virulence plasmid under these conditions. Under all tested conditions and at all time points, the number of bacteria that had lost the virulence plasmid was increased in Δ*parDE* cells, supporting the role of ParDE in virulence plasmid maintenance. These results were confirmed by observing EGFP-SctQ foci under nonsecreting conditions. The fluorescence construct is expressed from the virulence plasmid; foci-free cells therefore indicate loss of the virulence plasmid, which occurred in a large fraction of bacteria within 72 h and was again more pronounced in cells lacking ParE or ParDE (**Figure 4A**). In secreting bacteria lacking ParDE or overexpressing ParD, loss of the virulence plasmid could also be observed in real time in growth curves. Although this plasmid loss is most likely a stochastic event, the OD_600_ of these strains, but not wild-type bacteria, consistently started to increase about 6 h after induction of T3SS expression by a temperature shift to 37°C, indicating that plasmid loss from this time point alleviated the SAGI in an increasing fraction of the bacterial populations (**Figure 2C**). In line with this interpretation, no increase in OD_600_ was observed in the presence of arsenate, which selects for the presence of the virulence plasmid (**Figure 4B**).

### ParE is toxic for *Y. enterocolitica* and triggers an SOS response

**Figure 5:**
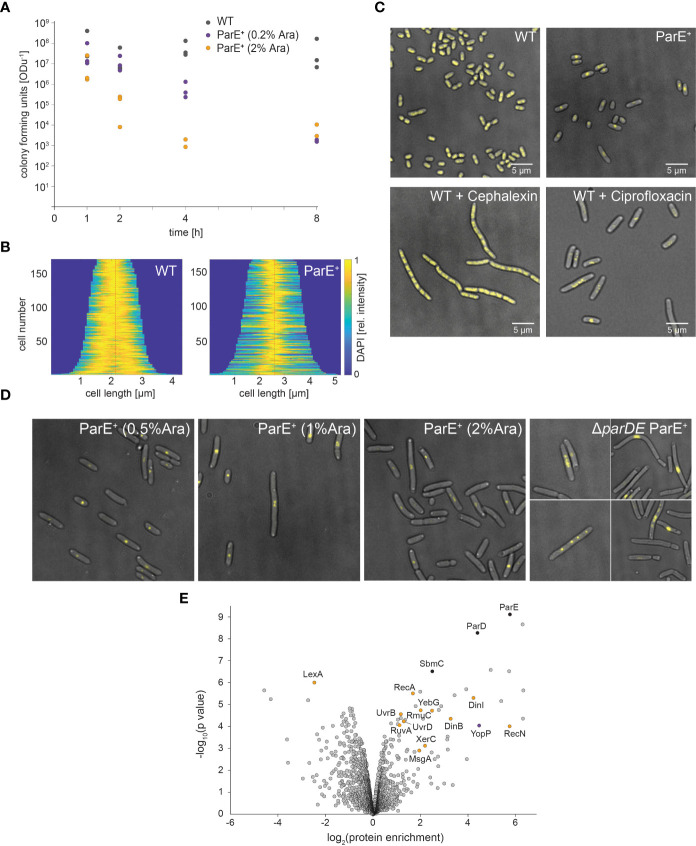
Effect of ParE expression on *Y. enterocolitica* viability, cell physiology, and proteome. **(A)** Viability of wild-type (WT, gray dots) and ParE-overexpressing cultures (ParE^+^). ParE expression was induced by 0.2% (purple dots) and 2% arabinose (orange dots). The expression of ParE toxins led to decreased cell viability (colony forming units per ODu, the equivalent of 1 ml culture at OD_600_ of 1). **(B, C)** DAPI staining visualizes DNA in wild-type (WT) *Y. enterocolitica* as well as bacteria additionally expressing ParE (ParE^+^) and treated with cephalexin or ciprofloxacin. **(B)** Demographs of DAPI distribution in WT and ParE^+^ strains; **(C)** representative images; **(D)** effect of ParE overexpression on DNA distribution; DAPI staining to visualize the DNA. ParE expression leads to highly compact DNA in the cell center in some DNA-free cells. In bacteria lacking ParD (right), expression of ParE (induced by 0.2% arabinose) resulted in bacteria with compact DNA in the cell center, as well as the formation of multiple foci and DNA-free cells. **(E)** Comparison of the proteome of non-secreting wild-type and ParE expressing cells. Proteins were quantified by LFQ mass spectrometry based on three biological replicates. The enrichment of proteins upon additional ParE expression and the statistical significance are marked in the volcano plot; selected proteins are indicated; see also **Supplementary Table 4** for details.

To observe whether ParE is bacteriostatic or whether it is also toxic and leads to cell death, we determined the number of viable cells in cultures with additional ParE expression from the plasmid. Expression of ParE decreased the number of viable cells in the cultures in a concentration-dependent manner (**Figure 5A**). While this effect occurred within less than 4 h, a low fraction (0.01%–0.1%) of bacteria remained viable over extended periods at increased ParE levels (**Figure 5A**).

ParE was shown to act as a gyrase inhibitor in *Escherichia coli* (Jiang et al., 2002), *Vibrio cholera* (Yuan et al., 2010), and *Mycobacterium tuberculosis* (Gupta et al., 2016). We, therefore, investigated the effect of ParE on the cellular distribution of DNA. DAPI staining of bacteria expressing ParE revealed strong compaction of the DNA, often at the center of the bacteria (**Figures 5B, C**). Further increases in ParE levels led to the loss of DNA in some cells (**Figure 5D**), in line with the decreasing number of viable cells in cultures after ParE expression. DNA localization of ParE-overexpressing bacteria was similar to treatment with the DNA gyrase inhibitor ciprofloxacin but not with cephalexin, which inhibits cell division but not DNA replication (**Figure 5C**), indicating that ParE directly acts on the DNA rather than cell division itself. Expression of ParE in the ParD-free cell background caused even stronger DNA compaction and some cells with several DAPI foci (**Figure 5D**), similar to ciprofloxacin-treated bacteria (**Figure 5C**).

To further characterize the influence of the toxin ParE on the bacteria, we analyzed its impact on the proteome by mass spectrometry-based LFQ (**Figure 5E**). Induction of ParE expression led to the upregulation of its antagonist, ParD. This could be due to the upregulation of expression or stabilization through complex formation with ParE (Dubiel et al., 2018). The protein SbmC was upregulated, which itself is a gyrase inhibitor. In the presence of gyrase-targeting toxins, however, it protects DNA (Chatterji et al., 2003), which is compatible with the role of ParE as a gyrase inhibitor. Taken together, these results demonstrate a strong effect of ParE on SOS response-associated proteins, especially proteins involved in DNA damage.

## Discussion

The virulence of *Yersinia* requires the presence of a 70-kb plasmid called the plasmid for *Yersinia* virulence (pYV) in *Y. enterocolitica* (Portnoy and Falkow, 1981). The presence of the virulence plasmid is essential for pathogenicity in *Yersinia* (Gemski et al., 1980; Zink et al., 1980; Ferber and Brubaker, 1981), and recent studies showed that the copy number of the virulence plasmid is tightly regulated in *Y. pseudotuberculosis*, which increases virulence (Wang et al., 2016). Among other virulence factors such as resistance and adhesion factors, these virulence plasmids mainly contain genes encoding the T3SS machinery, T3SS-exported effector proteins called Yops (*Yersinia* outer proteins), and specific Yop chaperones. Moreover, in close proximity to the *yopE* effector gene, two genes with homology to ParDE TA systems are encoded. Despite their short length and inconsistent annotation, we found that these genes are conserved within pathogenic *Yersinia* species (**Supplementary Figure 2**). While the primary sequence is only conserved within *Yersinia* and differs from well-studied ParDE of other species, the secondary structure is conserved (**Supplementary Figure 2**), which is commonly the case for TA systems (Rawlings, 1999; Deane and Rawlings, 2004; Ames et al., 2019; Kamruzzaman and Iredell, 2019).

Even though *parDE* is genetically closely linked with the T3SS, in particular the effector YopE, which is expressed from an operon overlapping with *parDE* (**Figure 1**), and secreting *Y. enterocolitica* cells have a similar phenotype to ParE overexpressing cells, ParDE is not the basis of the SAGI (**Figure 2**). Thus, the reason why the growth of *Y. enterocolitica*, as well as many other species, is inhibited during secretion remains unknown. Since it was reported that in absence of the HigA antitoxin, mRNA levels of the *P. aeruginosa* T3SS components were upregulated (Li et al., 2016), we tested for an impact of ParDE levels on the *Y. enterocolitica* proteome. However, while the level of proteins characteristically upregulated in an SOS response was increased, neither the absence nor overexpression of ParD or ParE had an impact on the expression of T3SS components in *Y. enterocolitica* (**Figure 5D**; **Supplementary Table 4**).

However, the ParDE TA system has an indirect influence on the T3SS by contributing to the maintenance of the virulence plasmid, especially under conditions where the T3SS is expressed and active (**Figure 3**). At 28°C, the frequency of loss of the virulence plasmid was low, even in the absence of ParDE. This can be explained by the lower burden of the plasmid, as most T3SS genes are only expressed to a low degree at this stage. At 37°C and especially under secreting conditions, plasmid loss was more pronounced and occurred significantly faster in strains lacking the ParDE TA system (**Figures 2C, 4B**), but also upon additional expression of the ParE-antagonist ParD (**Figure 2C**). Despite its low stability, the additional antitoxin ParD was apparently able to neutralize the remaining ParE after plasmid loss, lifting the selection pressure exerted by the TA system.

The relatively rapid loss of the virulence plasmid at 37°C under nonsecreting conditions, even in the presence of ParDE, is at first glance unexpected, given the essential role of the T3SS in establishing an acute infection. However, a population of pYV-free cells might well be beneficial for *Yersinia* during infection, similar to *Salmonella enterica* and *Pseudomonas aeruginosa*, which have T3SS-positive and T3SS-negative subpopulations (although based on a different mechanism in *Salmonella* SPI-1) (Rietsch and Mekalanos, 2006; Sturm et al., 2011; Rundell et al., 2016). pYV-free and therefore T3SS-negative *Yersinia* might divide more quickly under conditions where the T3SS is not required for defense against the immune system, such as in micro-colonies. In line with this interpretation, *Y. pestis* can induce a local anti-inflammatory state in pulmonary tissues that allows for the growth of normally avirulent bacteria, such as bacteria that have lost their virulence plasmid (Price et al., 2012). Absence of the T3SS might also prevent immune responses, e.g., against the needle tip protein, which is a long-known potent antigen (V antigen, (Motin et al., 1994)); however, it has also been suggested that replicating avirulent bacteria could act as a decoy for the immune system (Tsai and Coombes, 2019; Martyn et al., 2022). Alternatively, loss of the virulence plasmid might occur in intracellular *Y. enterocolitica*.

The precise circumstances under which bacteria might benefit from a loss of the virulence plasmid are difficult to determine, given the many unknowns in the infection of different *Yersinia* species. However, complete loss of the virulence plasmid in a population would be an evolutionary dead end, which would at least prevent the re-infection of further hosts. It is therefore likely that within the host, additional factors preserve plasmid maintenance. As quorum sensing was shown to affect plasmid stability (Ng et al., 2018), cell density *in vivo* might limit the loss of the virulence plasmid. In *Shigella flexneri* and *Shigella sonnei*, which also encode the T3SS on large virulence plasmids, several TA systems with different activity profiles have been found to contribute to the maintenance of the plasmid (McVicker and Tang, 2017; Pilla et al., 2017; Pilla and Tang, 2018; Martyn et al., 2022). However, no additional virulence plasmid-associated TA systems have been described in *Yersinia* so far.

The role of TA systems in the maintenance of plasmids is based on the function of the toxin that is released from the antitoxin-toxin complex after plasmid loss. In *Y. enterocolitica*, ParE is toxic (**Figure 5**), and increased ParE expression results in growth inhibition (**Figure 2**). Furthermore, ParE leads to an elongated cell morphology. All described effects are common for ParE-like proteins from different species (Roberts et al., 1994; Jiang et al., 2002; Fiebig et al., 2010; Kamruzzaman and Iredell, 2019), indicating a functional homology despite the low sequence conservation (**Supplementary Figure 2**). In cultures overexpressing ParE, the number of viable cells drops quickly but then remains stable at 0.01%–0.1% (**Figure 5A**). Such a pool of viable bacteria is not uncommon for bactericidal ParE (Ames et al., 2019). The remaining cells might be persister cells with strongly reduced growth and division (Shah et al., 2006; Kamruzzaman and Iredell, 2019).

The toxin ParE inhibits gyrases similar to the toxin CcdB and fluoroquinolone antibiotics like ciprofloxacin (Bernard and Couturier, 1992; Bernard et al., 1993; Jiang et al., 2002). Gyrases relieve topological stress during DNA replication and transcription. ParE of different bacterial species and fluoroquinolone antibiotics differ in the intensity of their effects (Ames et al., 2019). A key aspect seems to be the target region within the gyrase (Bernard and Couturier, 1992; Bahassi et al., 1995; Yuan et al., 2010; Gupta et al., 2016). In *Y. enterocolitica*, ParE expression results in highly compacted DNA (**Figure 5B**), an effect similar to ParE of *Caulobacter crescentus* (Ames et al., 2019). In antitoxin-free *Y.enterocolitica* cells, the DNA was sometimes separated into multiple foci (**Figure 5D**), indicating potent neutralization of the toxin by ParD.

In this study, we have characterized the role of the ParDE TA system in the virulence of *Yersinia enterocolitica*. We found that, despite the genetic overlap between *parDE* and the operon of a T3SS effector (*yopE-orf_98_*) and phenotypic similarities between secreting and ParE-expressing *Y. enterocolitica*, ParDE is not the basis of the SAGI. The toxin-antitoxin ratio is not altered in secreting bacteria; conversely, ParDE does not directly influence the assembly or activity of the T3SS. However, ParDE strongly increases the fraction of T3SS bacteria by maintaining the presence of the *Yersinia* virulence plasmid, especially under conditions and in time ranges relevant to acute infections (first 48 h at 37°C). This is the first characterization of a TA system involved in the maintenance of the *Yersinia* virulence plasmid, highlighting the double-edged role of the T3SS and the resulting tight control of its presence and activity in bacterial infections.

## Material and methods

### Bacterial strains

Bacterial strains and plasmids used in this study are summarized in **Supplementary Table S5**. All used strains are based on two strain backgrounds of *Y. enterocolitica*: wild-type strain MRS40 and ΔHOPEMTasd. ΔHOPEMTasd lacks the main virulence effectors (YopH, O, P, E, M, and T) as well as the aspartate-beta-semialdehyde dehydrogenase gene (*asd*) (Kudryashev et al., 2013). The strain is consequently avirulent and is classified as biosafety level 1. Furthermore, it is auxotrophic for the cell wall component diaminopimelic acid (Dap).

Strains used to jam the needle carry a pBAD-based plasmid (pAD642) expressing a C-terminal EGFP fusion to a long synthetic effector protein based on the *Y. enterocolitica* effector YopO, of which amino acids 20–77 were replaced by a FLAG epitope for detection and which was extended by an unstructured region of the protein SctP (pBAD::YopO_1-19_-FLAG-YopO_78-728_-SctP_308-381_-EGFP). Plasmid pAD633, coding for the equivalent construct without EGFP, served as a control.

### Bacterial cultivation

*Y. enterocolitica* strains were cultivated at 28°C in Brain Heart Infusion (BHI) medium (3.7% w/v) containing nalidixic acid (Nal) (35 µg/ml) and Dap (60 µg/ml) for ΔHOPEMTasd-based strains. For secretion assays, fresh cultures supplemented with MgCl_2_ (20 mM), glycerol (0.4% v/v), and EGTA (5 mM) for secreting conditions (secreting medium) or CaCl_2_ (0.75 mM) for nonsecreting conditions (nonsecreting medium) were inoculated to on OD_600_ of 0.1–0.15 and incubated shaking at 28°C for 1.5–2 h. Expression of the T3SS components from the *yop* regulon was induced by a temperature shift to 37°C; further analysis was performed after 150–180 min of shaking incubation at 37°C unless indicated otherwise. Ampicillin (0.2 mg/ml) was added to select pBAD-containing cells. Expression from the pBAD vector was induced by the addition of 0.2% l-arabinose, unless another concentration is indicated.

### Sequence analysis

A comparison of the genetic environment of *yopE-orf98* and *parDE* was analyzed for *Y. enterocolitica* pYVe227 (NCBI sequence identifier AF102990.1) and pYVe8081 (AM286416.1), *Y. pestis* CO92 pCD1 (AL117189.1) and biovar Microtus str. 91001 pCD1 (AE017043.1), and *Y. pseudotuberculosis* PB1/+ pYPTS01 (CP001049.1) and strain IP2666 pYV2666 (NZ_KT307967.1).

The following sequences were analyzed for ParD: WP_016677119.1 (*Yersinia pestis*), WP_012414146.1 (*Yersinia pseudotuberculosis*), EBI1835039.1 (*Salmonella enterica*), NHW59268.1 (*Escherichia coli*), WP_096052511.1 (*Caulobacter crescentus*), and WP_003409899.1 (*Mycobacterium tuberculosis*). For ParE, the following sequences were analyzed: WP_038930941.1 (*Yersinia pestis*), WP_057532641.1 (*Yersinia pseudotuberculosis*), WP_001143647.1 (*Salmonella enterica*), WP_011205808.1 (*Escherichia coli*), WP_096034173.1 (*Caulobacter crescentus*), and WP_003411124.1 (*Mycobacterium tuberculosis*). Alignment based on the amino acid sequence was performed by UniProt (Bateman et al., 2021). The secondary structure was predicted by using PSIPRED (Jones, 1999).

### Growth curve experiments

All growth curves were performed on a plate reader (Tecan Infinite 200 Pro photometer). Nonsecreting or secreting mediums were inoculated to an OD_600_ of 0.03 with overnight cultures. The cultures were incubated by shaking at 28°C. After 90 min, l-arabinose was added to the pBAD-containing cultures to induce expression. Cultures were distributed in quadruplicates into a 96-well microplate (150 µl/well). The measurement of growth curves was started immediately after arabinose addition in a plate reader at 37°C for 16 h. The conversion of the measured absorbance into OD_600_ was based on a calibration using the same culture volume and microtiter plate. To test for virulence plasmid loss, 2 mM arsenate was added before starting the measurement, where indicated.

### Secretion assay and protein analysis

For secretion assays, cultures were grown for 90 min at 28°C. At this time point, l-arabinose was added where required, and cultures were incubated for the indicated time at 37°C. Bacteria from a 2-ml culture were collected (15,000×*g*, 10 min, 4°C). To precipitate proteins in the supernatant, 1.8 ml of supernatant was mixed with 0.2 ml of trichloroacetic acid (final: 10%) and incubated over night at 4°C. Proteins were collected (15,000×*g*, 15 min, 4°C) and washed twice with ice-cold acetone (15,000×*g*, 5 min, 4°C). The pellet dried at room temperature for 1 h. To normalize the number of proteins to 0.6 OD units (ODu, equivalent to 1 ml of culture at OD_600_ of 0.6) in 15 µl of loading buffer, the pellet was resuspended in SDS-PAGE loading buffer (resuspension volume = OD_600_ * 45 µl) (SDS (2% w/v), Tris-HCl (0.1 M), glycerol (10% w/v), DTT (0.05 M), pH = 6.8). Total cell samples were prepared for western blot analyses by directly normalizing to 0.3 OD units in 15 µl of loading buffer (OD_600_ * 1.8 ml * 15 μl/0.3 ODu). All samples were heated for 10 min at 99°C before loading. Proteins were separated by SDS-PAGE on 15% acrylamide gels. For visualization, the gels were stained with InstantBlue (Expedeon, Heidelberg, Germany). For immunoblots, the proteins were blotted on a nitrocellulose membrane. Detection of the FLAG-tag was performed by using primary rabbit antibodies against FLAG (1:5000) (Rockland, Pottstown, PA, USA 600-401-383S) and secondary anti-rabbit antibodies conjugated with horseradish peroxidase (1:10,000) (Sigma-Aldrich, Darmstadt, Germany A8275). The immunoblot was visualized using an ECL chemiluminescence substrate (Pierce, Waltham, MA, USA) on a LAS-4000 Luminescence Image Analyzer (Fujifilm, Ratingen, Germany).

### Infection assay

Infection was measured by luciferase complementation mediated through translocation of the small part of the NanoLuc luciferase (HiBiT, fused to the T3SS secretion signal YopE_1–53_) expressed from a pBAD vector in *Y. enterocolitica* into eukaryotic target cells (HeLa cells expressing the complementary large NanoLuc fragment (LgBiT)). The full-length luciferase then catalyzes a reaction, resulting in a luminescence signal (Lindner et al., 2020; Westerhausen et al., 2020). Eukaryotic cells were seeded into a microplate (20,000 cells/well) and incubated overnight at 37°C with 5% CO_2_. Before starting the experiment, the medium was replaced by a colorless medium (120 μl/well) (RPMI medium 1640, with l-glutamine, without Phenol Red, Gibco, Carlsbad, CA, USA) containing 60 µg/ml Dap. Bacterial cells were inoculated from overnight culture into a nonsecreting medium (80 µl into 5 ml). After 90 min of incubation at 28°C, cultures were shifted to 37°C and incubated for an additional 3 h to induce T3SS assembly. After incubation, 0.5 OD units were collected (2,400×*g*, 4 min) and washed as well as resuspended in 1 ml phosphate-buffered saline (PBS) (8 g/l NaCl, 0.2 g/l KCl, 1.78 g/l Na_2_HPO_4_*2H_2_O, 0.24 g/l KH_2_PO_4_, pH 7.4) containing 0.2% l-arabinose and 5 mM Dap. For infection, 11.2 µl of bacterial suspension (corresponding to 140 bacteria per target cell) and 80 µl of Luciferase working solution (Nano-Glo Luciferase Assay Substrate in Luciferase Assay Buffer, 1:50, PROMEGA, Madison, CO, USA) containing 0.5% l-arabinose were added to eukaryotic cells. The measurement of the luminescence started directly on a prewarmed plate reader at 37°C.

### Toxicity test

Overnight cultures were inoculated into a nonsecreting medium to an OD_600_ of 0.1. After 1 h of incubation at 28°C, cultures were shifted to 37°C. After 1, 2, 4, and 8 h, samples were taken, and 50 µl of serial dilutions were plated on solid LB agar. The number of viable bacteria in 1 ml culture at an OD_600_ of 1 was determined by the number of colonies, taking into account the dilution factor, the plated volume, and the OD_600_ of the culture.

### Microscopy

For microscopy, cells were inoculated from overnight cultures into fresh nonsecreting or secreting medium and incubated at 28°C. After 90 min, supplements were added as indicated (l-arabinose, 35 μg/ml cephalexin, 20 μg/ml ciprofloxacin), and cultures were shifted to 37°C. At different time points, samples were directly analyzed under a microscope or first stained with DAPI.

For DAPI staining, 400 µl of culture was collected after 2.5 h at 37°C (2,600×*g*, 1 min, 37°C). The cells were resuspended in 40 µl of nonsecreting medium and 10 µl of DAPI (2.5 µg/ml). After staining for 2 min at 37°C, the cells were pelleted (2,600×*g*, 1 min, 37°C) and resuspended in 200 µl of nonsecreting medium.

For microscopy, 2 µl culture were spotted on agarose pads (1.5% (w/v) agarose (Sigma-Aldrich, Darmstadt, Germany) in minimal medium (100 mM 2-[4-(2-hydroxyethyl)piperazin-1-yl]ethane-1-sulfonic acid (HEPES) at pH 7.2, 5 mM (NH_4_)_2_SO_4_, 100 mM NaCl, 20 mM sodium glutamate, 10 mM MgCl_2_, 5 mM K_2_SO_4_, 50 mM 2-(*N*-morpholino)ethanesulfonic acid (MES), 50 mM glycine)). Microscopy was performed on an inverse epifluorescence microscope (Deltavision Elite Optical Sectioning Microscope, Applied Precision, Mörfelden-Walldorf, Germany), equipped with a UPlanSApo ×100/1.40 oil objective (Olympus, Center Valley, CA, USA) and an EDGE sCMOS_5.5 camera. Cell morphology was observed by differential interference contrast (DIC). To visualize GFP fluorescence signals, a GFP filter set (excitation: 490/20 nm, emission: 525/30 nm) was used with a 0.2-s exposure time. For imaging of DAPI-stained DNA, a DAPI filter set (excitation: 390/18 nm, emission: 435/48 nm) was used with a 0.2-s exposure time. Images were subsequently deconvolved using softWoRx 7.0.0 (standard “conservative” settings). The images were analyzed with ImageJ-FIJI (ImageJ 1.51f/1.52i/1.52n) to measure cell length or to create overlays (Schindelin et al., 2012). Demographics were created by using BacStalk (Hartmann et al., 2020).

### Plasmid stability assay

The plasmid stability assay is based on arsenate resistance encoded on the pYV plasmid (Iriarte and Cornelis, 1998). The overnight culture was inoculated into BHI (for 28°C assays) or secreting and nonsecreting medium, as described above, for 37°C assays, respectively (1:1,000 dilution). Each medium was supplemented with Dap and Nal. Over 5 days, the cultures were repeatedly diluted in fresh medium every 12 h (1:1,000 dilution). At certain time points, samples were taken before the transfer. Bacteria were serially diluted and plated on solid LB agar containing Dap and Nal (nonselective for virulence plasmid). For each condition, 100 colonies were streaked on LB agar containing 2 mM arsenate, Dap, and Nal (selective for virulence plasmid).

### Proteome and secretome analysis by mass spectrometry

The overnight cultures were inoculated into nonsecreting to OD_600_ = 0.12 or secreting medium to OD_600_ = 0.15 and incubated for 90 min at 28°C. l-Arabinose was then added to the pBAD-containing strains (0.2%, unless indicated differently), and the cultures were switched to 37°C. After 2.5 h at 37°C, the total cell samples for proteome analysis were taken and normalized for their OD_600_. The cells were washed three times with ice-cold PBS (15,000×*g*, 10 min, 4°C) and resuspended in 300 μl lysis buffer (2% sodium lauroyl sarcosinate (SLS), 100 mM ammonium bicarbonate). Samples were then heated for 10 min at 90°C. The number of proteins was determined by bicinchoninic acid protein assay (Thermo Scientific). Proteins were reduced with 5 mM Tris(2-carboxyethyl) phosphine (Thermo Fisher Scientific, Waltham, MA, USA) at 90°C for 15 min and alkylated using 10 mM iodoacetamide (Sigma-Aldrich, Darmstadt, Germany) at 20°C for 30 min in the dark. For tryptic digestion 50 µg of protein was incubated in 0.5% SLS and 1 µg of trypsin (Serva, Heidelberg, Germany) at 30°C overnight. After digestion, SLS was precipitated by adding a final concentration of 1.5% trifluoroacetic acid (TFA, Thermo Fischer Scientific, Waltham, MA, USA). Peptides were desalted by using C18 solid phase extraction cartridges (Macherey-Nagel, Düren, Germany). Cartridges were prepared by adding acetonitrile (ACN), followed by equilibration with 0.1% TFA. Peptides were loaded on equilibrated cartridges, washed with 5% ACN and 0.1% TFA-containing buffer, and finally eluted with 50% ACN and 0.1% TFA. Peptides were dried in a rotating concentrator (Thermo Fischer Scientific, Waltham, MA, USA) and reconstituted in 0.1% TFA for liquid chromatography-mass spectrometry (LC-MS) analysis.

LC-MS analysis was performed on an Exploris 480 instrument connected to an Ultimate 3000 rapid-separation liquid chromatography (RSLC) nanoinstrument and a nanospray flex ion source (all Thermo Scientific). Peptide separation was carried out on a reversed-phase high-performance liquid chromatography (HPLC) column (75 μm × 42 cm) packed in-house with C18 resin (2.4 μm; Dr. Maisch GmbH). First, peptides were loaded on a C18 precolumn (preconcentration set-up). For total proteome analysis, peptide elution was performed in backflush mode with a separating gradient from 94% solvent A (0.15% formic acid) and 6% solvent B (99.85% ACN, 0.15% formic acid) to 25% solvent B over 65 min, followed by an increase of 25% to 35% of solvent B for additional 25 min at a flow rate of 300 nl/min. Label-free quantification (LFQ) datasets of total proteomes were performed in data-dependent acquisition (DDA) and data-independent acquisition (DIA) modes. For DDA analysis, a high-resolution MS 1 scan at a resolution of 60,000 (at m/z 200) with a scan range from 350 to m/z 1,650 was acquired, followed by MS/MS scans of the 20 most intense ions within 2 s (cycle 2s) at a resolution of 15,000. Charge state inclusion was set between 2 and 6. The ion accumulation time was set to 25 ms for MS and AUTO for MS/MS. The automatic gain control (AGC) was set to 300% for MS survey scans and 200% for MS/MS scans.

DDA-LFQ analysis was performed using MaxQuant version 1.6.17.0 (Cox and Mann, 2008) in standard settings using a protein database containing proteins of the closely related *Y. enterocolitica* strain W22703 (Fuchs et al., 2011) and of the pYVe227 virulence plasmid (GenBank entry AF102990.1). The statistical analysis of the MaxQuant LFQ data was performed on an updated SafeQuant R-script (Glatter et al., 2012; Ahrné et al, 2016) to routinely process MaxQuant “protein group” outputs.

For DIA analysis, the LC gradient was adjusted to 6%–25% buffer B for 40 min and an additional 20% to 35% of buffer B. DIA datasets were acquired using 45 windows with an isolation window of m/z 14 with m/z 1 overlap. MS scan resolution was set to 120,000 (MS1) and 15,000 (DIA) with a scan range of m/z 350–1,400 (MS1) and 320–950 precursor mass range (DIA). AGC target settings were 300% (MS1) and 3,000% (DIA) with a maximum ion injection time of 50 ms (MS1) and 22 ms (DIA).

DIA data were analyzed using DIA-NN version 1.8 (Demichev et al., 2020). The full tryptic digest was allowed with two missed cleavage sites and oxidized methionines and carbamidomethylated cysteines. Matching between runs and removing likely interferences were enabled. The neural network classifier was set to the single-pass mode, and protein inference was based on genes. The quantification strategy was set to any LC (high accuracy). Cross-run normalization was set to be RT-dependent. Library generation was set to smart profiling. DIA-NN outputs were further evaluated using R.

For the secretome analysis, 4 ml of the remaining cultures (used for proteome analysis) were pelleted (2,400×*g*, 5 min, 37°C) and OD-normalized in 4 ml of secreting or nonsecreting minimal medium with 0.4% casamino acids. The cultures were further incubated for 1 h at 37°C. After incubation, 2 ml cultures were centrifuged (15,000×*g*, 10 min, 4°C). 1.8 ml of the supernatant was mixed with 0.2 ml trichloroacetic acid (final: 10%) and incubated over night at 4°C to precipitate proteins within the supernatant. Proteins were collected (15,000×*g*, 15 min, 4°C) and washed two times with ice-cold acetone (15,000×*g*, 5 min, 4°C). The pellet was dried at room temperature for 1 h, resuspended in 300 µl of lysis buffer, and heated for 10 min at 90°C. Proteins of the secretome were treated identically to the proteins of the proteome (see above). Due to lower sample complexity, the LC-separating gradient length was reduced to 3,045 min. The MS data were acquired in DDA mode using identical settings as described before, except that the shorter gradient MS/MS spectra of the top 20 most intense precursors were recorded. The data analysis was performed using MaxQuant. Proteomics data have been deposited to the ProteomeXchange Consortium via the PRIDE (Perez-Riverol et al., 2022) partner repository with the dataset identifier PXD040518.

### RNA isolation and RT-PCR

One volume of cell culture was mixed with two volumes of RNAprotect Bacteria Reagent (Qiagen, Hilden, Germany). Cells were harvested by centrifugation and frozen in liquid nitrogen. Total RNA was purified with the RNeasy Mini Kit (Qiagen, Hilden, Germany). RNA concentration and purity were analyzed by measuring absorbance at 260 and 280 nm. RNA integrity was monitored by electrophoresis in a 1% agarose-TBE-gel containing 25 mM guanidium thiocyanate followed by ethidium bromide staining. To remove residual DNA, 10 µg of RNA was treated with 1 µl of TURBO-DNase (Thermo Fischer Scientific, Waltham, MA, USA) for 30 min. DNA removal was validated by PCR with the *yopE*- specific primers YopE-Fwd 5′-TCATCAATGGCCCACTCTGT-3′ and YopE-Rev 5′-TGCATGTATTTTGGCAGCGT.

To test whether *yopE* and *orf98* are co-transcribed, the following primers were used: YopE-ORF98-Fwd 5′-ATGAGATCAAAGGGCTGGGG-3′ and YopE-ORF98-Rev 5′-CGAGTGCTGCATCAATCCAT-3′. Co-transcription of *parD* and *parE* was analyzed with the primers ParDE-Fwd 5′-TAGCCTGAGTCACACCGAAA-3′ and ParDE-Rev 5′-CAGCGTGTTCAACATGTACG-3′. The strand-specific RT-PCR analysis was performed using the Brilliant III Ultra-Fast SYBR^®^ Green QRT-PCR Mastermix (Agilent), although the results were not evaluated quantitatively. The reactions sample contained 5 µl Master Mix (supplied), 0.1 µl DTT (100 mM, supplied), 0.5 µl Ribo-Block solution (supplied), 0.4 µl ultrapure water, 1 µl reverse primer (10 pmol/µl), and 2 µl RNA (20 ng/µl). The control samples contained 2 µl of total DNA (20 ng/µl; positive control for the PCR) or 2 µl of ultrapure water (negative control). After incubation for 10 min at 50°C (RT-step resulting in cDNA synthesis in the RNA-containing sample), the reverse transcriptase was inactivated at 95°C for 3 min. The samples were cooled down to 4°C, and the forward primer was added for PCR, which was started at 95°C for 1 min. The PCR program included 39 cycles of 3 s at 94°C, 5 s at 56°C, and 5 s at 60°C. In a control demonstrating that the used RNA is DNA-free, the RT-step was omitted. Both forward and reverse primers were added to the reaction mixture, which was then incubated at 95°C for 4 min, and PCR was conducted. The PCR products were analyzed by electrophoresis in a 10% polyacrylamide gel, which was stained with ethidium bromide.

For quantitative analysis of RNA levels by strand-specific reverse transcription followed by real-time PCR (qRT-PCR), the Brilliant III Ultra-Fast SYBR^®^ Green QRT-PCR Mastermix (Agilent) was used as described before. As a reference gene, we used a spike-in transcript (1 ng/µl *in vitro* transcript of the gene *rrp41* of *Sulfolobus solfataricus* (Li et al., 2021)), 1 ng of which was added to the resuspended bacteria at the first step of the RNeasy Mini Kit purification procedure. For *yopE* analysis, the abovementioned primers YopE-Fwd and YopE-Rev were used. Their primer pair efficiency (PE), estimated using twofold dilutions of the DNA-free RNA in qRT-PCR, was 2.05. For *parD* analysis, primers ParD-Fwd (5′-GTATCTTTCACCGCAGCAGA-3′) and ParD-Rev (5′-GCATTTGACAGGTTTTGTGG-3′) were used (PE: 2.03); for *parE* analysis, primers ParE-Fwd (5′-ATACTGAAAACGGCGCATGT-3′) and ParE-Rev (5′-TCTGAACTGGCTGATGAGGA-3′) were used (PE: 1.97); for analysis of the *parDE* co-transcript, primers ParDE-Fwd (5′-TAGCCTGAGTCACACCGAAA-3′) and ParDE-Rev (5′-CAGCGTGTTCAACATGTACG-3′) were used (PE: 2.18). For the spike-in reference transcript, primers Sso41-qPCR-Fwd (5′-GCATCCAAGGCACCTATCTC-3′) and Sso-41-qPCR-Rev (5′-GGAGGCGGCCATTAATGAAA-3′) were used (PE: 2.02). Primer pairs were designed using Primer3 (Untergasser et al, 2007). The qRT-PCR reactions were performed in a spectrofluorometric thermal cycler (BioRad, München, Germany). The Pfaffl formula was used to calculate fold changes in mRNA amounts (Pfaffl, 2001).

## Data availability statement

The datasets presented in this study can be found in online repositories. The names of the repository/repositories and accession number(s) can be found below: ProteomeXchange via the PRIDE database: PXD040518.

## Author contributions

SS performed the majority of the experiments and participated in experimental design, data analysis, and writing of the manuscript. RS performed the RT-PCR experiments and data analysis. FE performed the experiments for ParE overexpression proteomics analysis and secretion assays. TG performed the proteomics experiments and data analysis. EE-H participated in RT-PCR experimental design, supervision, and data analysis. AD designed and supervised the project and experimental design and participated in data analysis and writing of the manuscript. All authors participated in discussion of the results and the manuscript. All authors contributed to the article and approved the submitted version.

## Footnotes

1 The T3SS is at the core of both injectisomes and bacterial flagella (Erhardt et al., 2010; Diepold and Armitage, 2015). In this manuscript, “T3SS” refers to the injectisome-type, virulence-associated system. The common nomenclature of T3SS subunits (Hueck, 1998; Wagner and Diepold, 2020) is used in this manuscript where applicable.

2 Called LcrF in *Y. pseudotuberculosis* and *Y. pestis.*

3 In this context, the SAGI is also termed “low calcium response” or “growth restriction”
