# Supplementary material for: A ParDE toxin–antitoxin system is responsible for the maintenance of the Yersinia virulence plasmid but not for type III secretion-associated growth inhibition
Source: Front Cell Infect Microbiol. 2023 May 9;13:1166077. doi: 10.3389/fcimb.2023.1166077 (PMC10203498; doi:10.3389/fcimb.2023.1166077)
Supplement: Supplementary file 1 [file DataSheet_1.pdf]

Supplementary information to

A ParDE toxin-antitoxin system is responsible for maintenance of the *Yersinia* virulence plasmid, but not for type III secretion-associated growth inhibition

Saskia Schott[1], Robina Scheuer[2], Francesca Ermoli[1], Timo Glatter[1], Elena Evguenieva-Hackenberg[2], Andreas Diepold[1]

1: Max Planck Institute for Terrestrial Microbiology, Department of Ecophysiology,

Karl-von-Frisch-Str. 10, 35043 Marburg, Germany

2: Justus Liebig University Gießen, Department of Microbiology and Molecular Biology,

Heinrich-Buff-Ring 26-32, 35392 Gießen, Germany

Correspondence: Andreas Diepold, andreas.diepold@mpi-marburg.mpg.de, +49-6421-178302

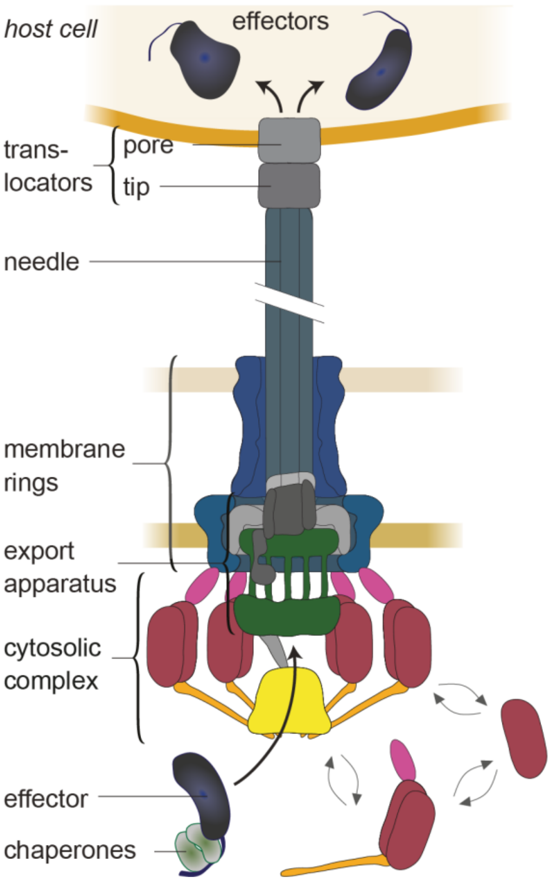

Supplementary Figure 1 – Schematic depiction of the bacterial type III secretion injectisome

A

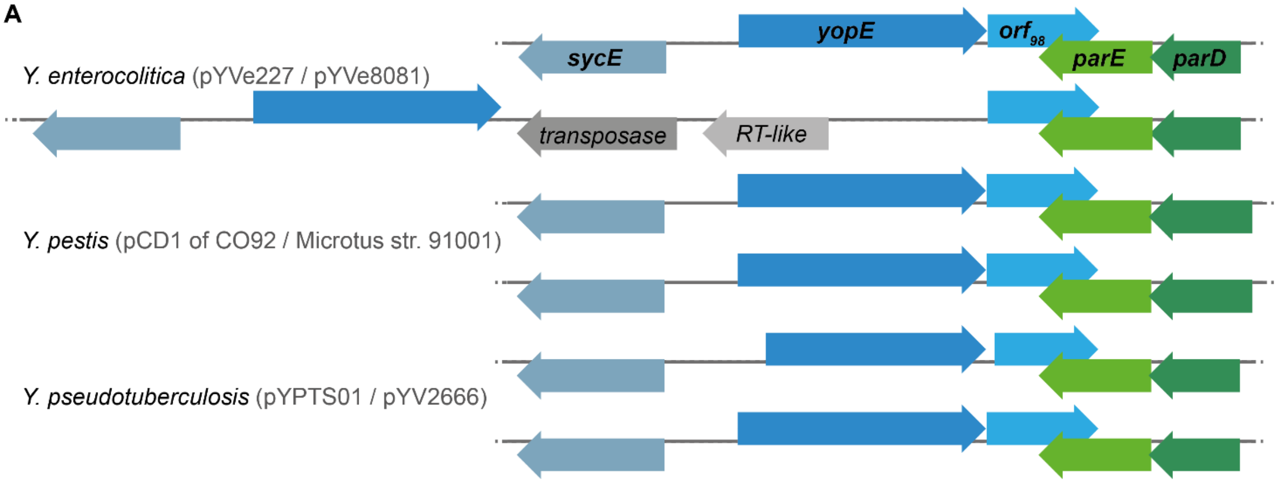

B

	sycE		*yopE*		*orf₉₈*		*parE*		*parD*		next gene	
Y. entero. pYVe227	-1381	392	-796	659	-135	296	0	302	295	242	681	203
Y. entero. pYVe8081	-2670	392	-2085	659	-135	296	0	302	295	242	681	320
Y. pestis CO92 pCD1	-1385	392	-799	659	-138	296	0	299	292	275	647	368
Y. pestis Microtus 91001 pCD1	-1385	392	-799	659	-138	296	0	299	292	275	678	203
Y. pseudotub. PB1/+ pYPTS01	-1385	392	-724	584	-117	275	0	299	292	242	678	200
Y. pseudotub. IP2666 pYV2666	-1385	392	-799	659	-138	296	0	299	292	242	700	292

C

β-strand
α-helix

Antitoxin ParD

```
Yersinia enterocolitica        --MARVTSVTLGEHLTGFVGEMIQSGRYGNISEVLRDALRLMEAREQRVQHVRDMVLAGT 58
Yersinia pseudotuberculosis    --MAHVTSVTLGEHLTGFVGEMIQSGRYGNISEVLRDALRLMEAREQRVQHVRDMVLAGT 58
Yersinia pestis                --MAHVTSVTLGEHLTGFVGEMIQSGRYGNISEVLRDALRLMEAREQRVQHVRDMVLAGT 58
Salmonella enterica            --MARVTSVTLGEHFNGFVGEMIESGRYGNTSEVLRDALRLMEAREQRLQNVREMVLAGV 58
Escherichia coli               --MARVTSVTLGEHFNGFVGEMINSGRYGNTSEVIRDALRMMEIREERIQIVRKMVLDGV 58
Caulobacter crescentus         MTTVSKRTVSLPAEQSRYIDELVATGTYASASEVVRAGVRALQERDAAVERWLRE----D 56
Mycobacterium tuberculosis     --MGKNTSFVLDEHYSAFIDGEIAAGRYRSASEVIRSALRLLEDRETQLRALREALEAGE 58

Yersinia enterocolitica        NAPVSHRLM-DEIFSAAVKDTS--V------------- 80
Yersinia pseudotuberculosis    NVPVSHRLM-DEIFSAAVKGTS--V------------- 80
Yersinia pestis                NVPVSHRLM-DEIFSAAVKDTS---------------- 79
Salmonella enterica            NAPVSQRSM-DEIFAAAVKNAS--V------------- 80
Escherichia coli               NSPESENNM-DDIFAKAEKDLN--V------------- 80
Caulobacter crescentus         VAPVYDAMLDDPGRAVAAKDVMASIRAHHALRLKAKPE 94
Mycobacterium tuberculosis     RSGSSTPFDFDGFLGRKRADAS--RGR----------- 83
```

sequence identity with ParD of *Y. enterocolitica*
96.25 %
97.47 %
83.75 %
70.00%
29.73 %
41.27 %

Toxin ParE

```
Yersinia enterocolitica        ---MYKLSELADEDIYNIASYTIRHFGVTQAKLYHENLAKVFELLAKNLELGAECNWICS 57
Yersinia pseudotuberculosis    ---MYKLSELADEDIYNIASYTIRRFGVTQAKLYHENLAKVFELLAKNPELGAECNWICS 57
Yersinia pestis                ------MSELADEDIYNIASYTIRHFGVTQAKLYHENLAKVFELLAKNPELGAECNWICS 54
Salmonella enterica            -MPTYKLSELADEDIFAIVRYTIQNFGVAQARSYHTDLTRVLELLAQNPELGSECNWICT 59
Escherichia coli               -MTAYILTAEAEADLRGIIRYTRREWGAAQVRRYIAKLEQGIARLAAGEGPFKDMSELFP 59
Caulobacter crescentus         -MKPYRLSRRAKADLDDIWTYSEQRWGVEQAADYARELQATIEMIAEHPGMGQPDENLRA 59
Mycobacterium tuberculosis     MTRRLRVHNGVEDDLFEAFSYYADAAPDQIDRLYNLFVDAVTKRIPQAPNAFAPL---FK 57

Yersinia enterocolitica        DMRRFQYKK--HGIYYI-TLSNDILISRVLHQSIDINAQDFPEYE----- 99
Yersinia pseudotuberculosis    DIRRFQYKK--HGIYYI-TLSNDILISRVLHQSIDIDVQDFPEHE----- 99
Yersinia pestis                DIRRFQYKK--HGIYYI-TLSNDILISRVLHQSIDIDVQDFPEHE----- 96
Salmonella enterica            GMRRFQFKK--HGIYLM-VLEEGILVSRVLHHSIDIDAQDFPE------- 99
Escherichia coli               ALRMARCEH--HYVFCLPRAGEPALVVAILHERMDLMTRLADRLKG---- 103
Caulobacter crescentus         GYRRCASGS--HVVFYR-V-GVRVEIIRVLHQSMNARAHLG--------- 96
Mycobacterium tuberculosis     HYRHIYLRPFRYYVAYR-TTDEAIDILAVRHGMENPN-AVEAEISGRTFE 105
```

sequence identity with ParE of *Y. enterocolitica*
93.94 %
93.75 %
69.79 %
no significant similarity
30.77 %
no significant similarity

Supplementary Figure 2 – Conservation of gene arrangements of the *yopE*/*parDE* region on the virulence plasmids of pathogenic *Yersinia* species and of the ParD/E secondary structure

(**A**, **B**) Analysis of the genetic environment of the *yopE*-*orf₉₈* and *parDE* region in the virulence plasmids of *Y. enterocolitica* pYVe227 and pYVe8081, *Y. pestis* CO92 pCD1 and biovar Microtus str. 91001 pCD1, *Y. pseudotuberculosis* PB1/+ pYPTS01 and strain IP2666 pYV2666. **(A)** Graphic depiction of genes; RT stands

for reverse transcriptase. **(B)** Predicted start sites with respect to the start codon of the *parE* toxin (green, left) and lengths in base pairs (blue, right) for the indicated genes. *Y. enterocolitica* pYVe8081 additionally contains a transposase gene (start -1385 / length 425 bp) and a reverse transcriptase-like gene (start -892 / length 557 bp). The genes downstream *parD* (next gene) are annotated as transposase remnants or pseudogenes. (**C**) ParD and ParE share low sequence identity except within the *Yersinia* family, but share a common predicted secondary structure (ParD: β1 α1 α2 α3; ParE: β1 α1 α2 β2 β3 β4). Sequences are aligned based on the amino acid sequence identity.

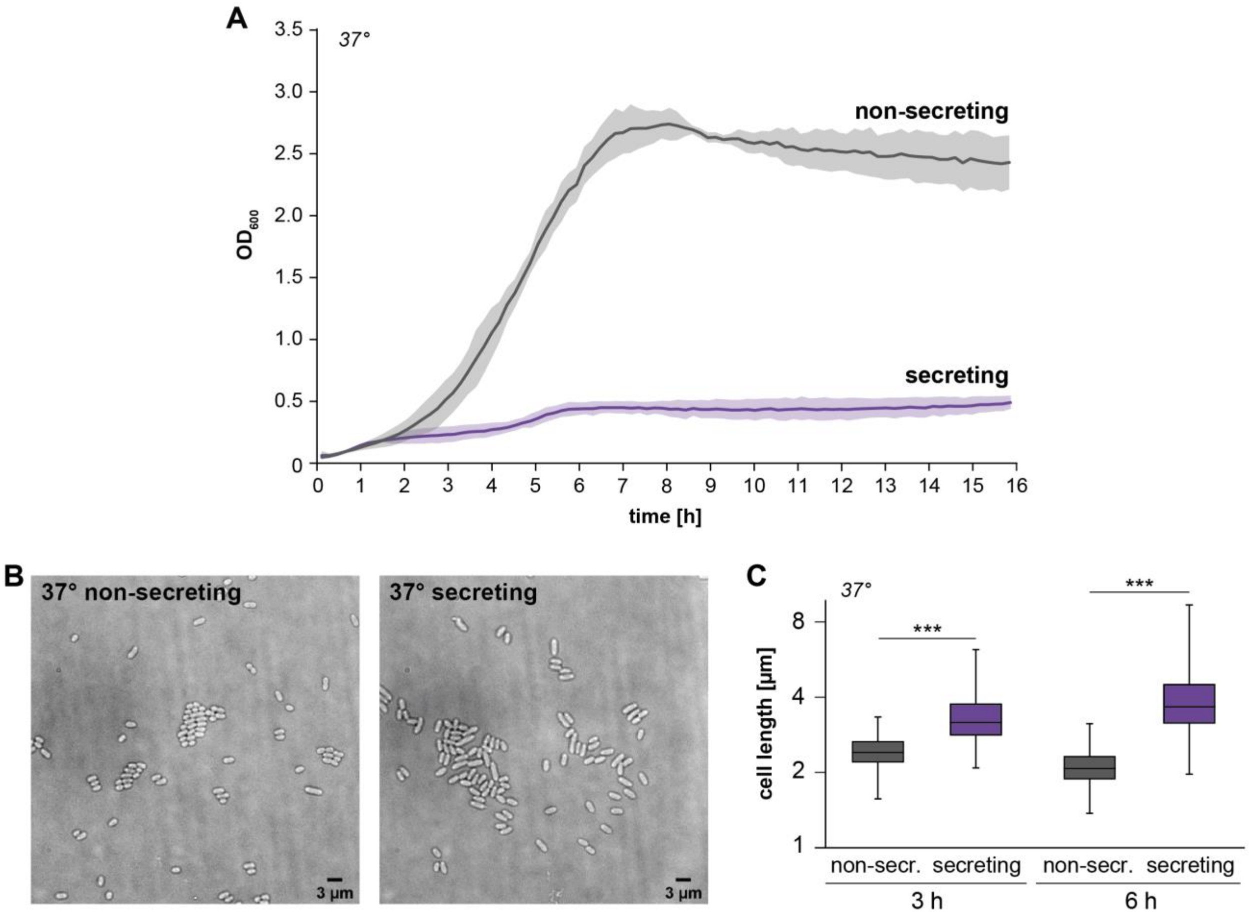

Supplementary Figure 3 - The type III secretion-associated growth inhibition in *Yersinia enterocolitica*

(A) Development over time of the optical density at 600 nm (OD_{600}) of cultures of secreting (purple) and non-secreting (grey) wild-type *Y. enterocolitica*, measured in a 96-well format in a shaking plate reader at 37°C. Average (solid lines) and standard deviation (shaded area) of three independent biological replicates, measured in technical quadruplicates. **(B, C)** Effect of secretion on cell morphology. Cell length values of 100 cells per biological replicate (n=3) are shown as box plots on a logarithmic scale. The box extends from the lower to the upper quartile. The median is indicated by a vertical line, whiskers indicate minimal and maximal values. ***p<0.001 in a two-tailed homoscedastic t-test.

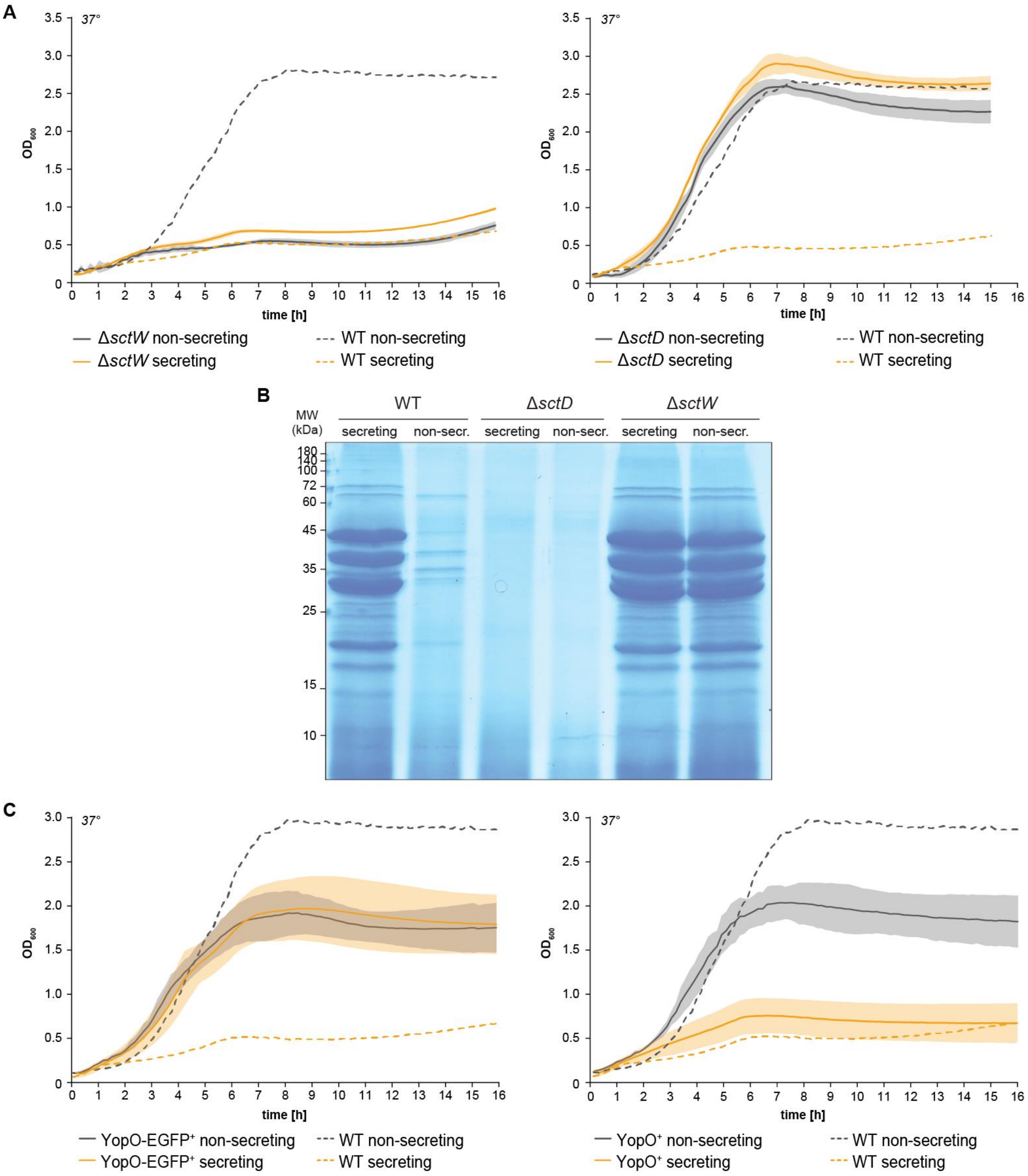

Supplementary Figure 4 – Growth inhibition is caused by actual protein secretion, rather than presence of a functional T3SS or external conditions

(A) Development of OD_{600} over time in *Y. enterocolitica* with a non-functional T3SS (deletion of the IM component SctD, Δ*sctD*) as well as "calcium-blind" *Y. enterocolitica* that secrete irrespective of cell contact

or Ca^{2+} levels (deletion of the gatekeeper protein SctW, Δ*sctW*). **(B)** Secretion assay for strains used in (A). Proteins secreted by $3*10^8$ bacteria in the indicated conditions were precipitated by addition of 10% TCA, separated by SDS-PAGE analysis and stained with Coomassie Brillant Blue. *n*=1, for additional strain characterization see (Boland *et al*, 1996; Diepold *et al*, 2017). **(C)** Development of OD_{600} over time in wild-type *Y. enterocolitica* expressing the T3SS effector YopO-EGFP, which obstructs the secretion channel, or the non-obstructing YopO (see main text for details). In all experiments, OD_{600} was measured in a shaking plate reader for 16 h at 37°C. Average (solid lines) and standard deviation (shaded area) of three independent biological replicates, measured in technical quadruplicates with wild-type strains serving as controls (WT, dashed lines).

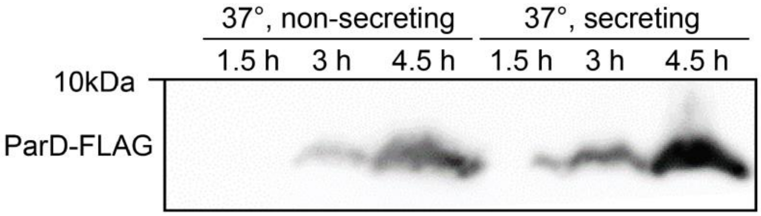

Supplementary Figure 5 – Expression of ParD-FLAG over time

Western blot anti-FLAG of total cellular proteins of showing expression of ParD (expected molecular weight = 8.8 kDa) upon induction with 0.2% arabinose in the indicating conditions, serving as expression control for Fig. 2CD. Representative image, *n*=3.

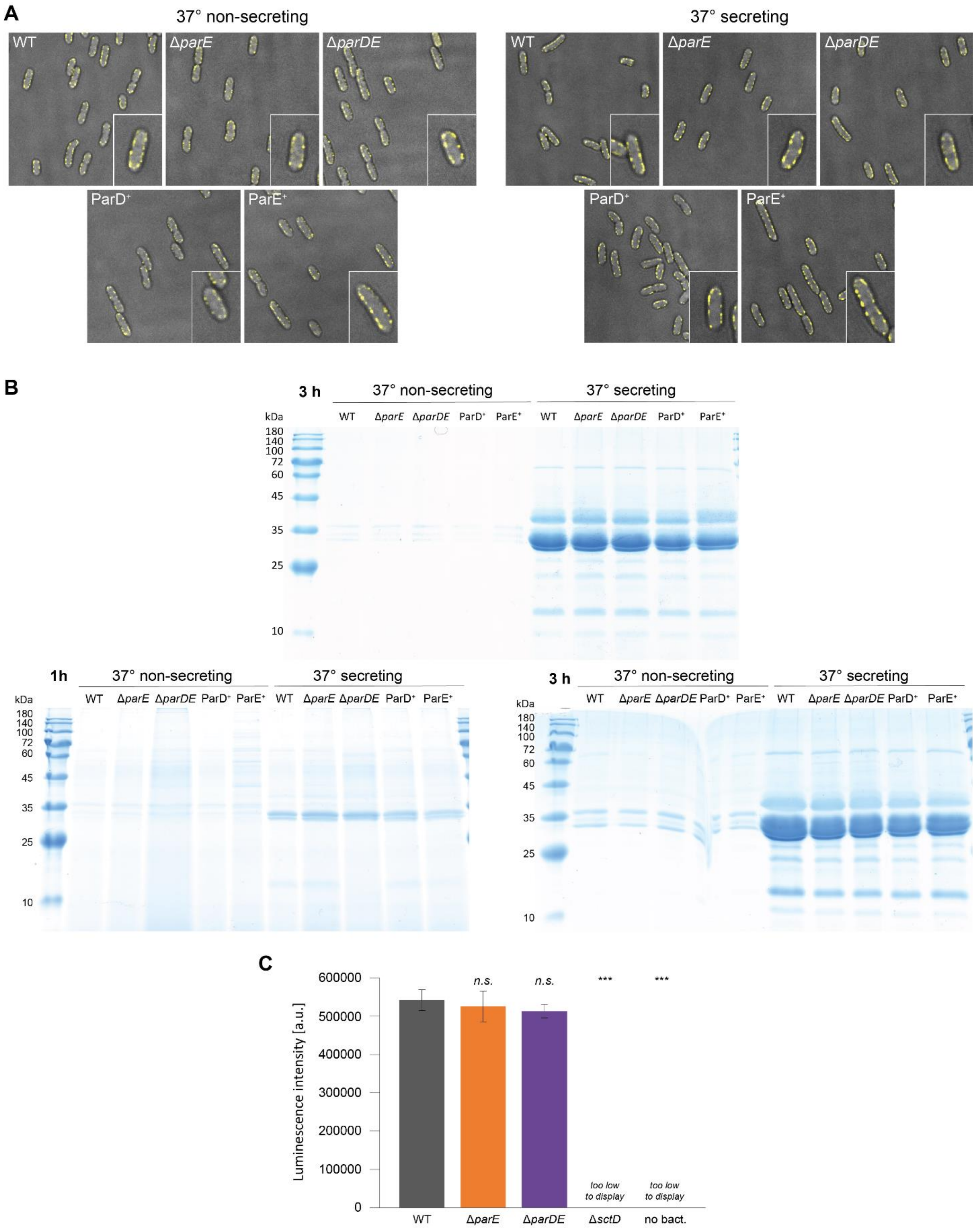

Supplementary Figure 6 – ParDE does not affect T3SS assembly and activity

(A) Overlay of the fluorescence signal of EGFP-SctQ, expressed from its native genetic localization (yellow) with corresponding DIC images (grey). After 3 h at 37°C under non-secreting (left) or secreting conditions

(right), bacteria were imaged. EGFP-SctQ localizes in foci at the membrane. The fluorescence pattern was not affected in cells missing the gene for the toxin *parE* (Δ*parE*) or the full *parDE* operon (Δ*parDE*). Moreover, ParD and ParE expression from an arabinose-inducible promotor had no apparent effect on the localization of EGFP-SctQ. Images are representative of three biological replicates. **(B)** Secretion assay visualizing secreted proteins within the culture supernatant. Samples were taken after the indicated incubation periods at 37°C. The influence on secretion was tested for wild-type (WT), a *parE* (Δ*parE*) and *parDE* (Δ*parDE*) deletion background and for cells with additional ParD and ParE expression. The amount of secreted proteins was not affected under the tested conditions. Gel images are representative for three biological replicates. **(C)** Effector translocation into HeLa cells by the indicated bacterial strains was visualized by complementation of luciferase activity by T3SS-dependent translocation of $YopE_{1\text{-}53}$-HiBiT. Strains lacking the integral IM component SctD (Δ*sctD*) and wells without bacteria (no bact.) were used as controls. The values represent the luminescence intensity measured one hour after the bacteria were added to the eukaryotic cells. Error bars indicate the standard deviation of three replicates. ***, $p<0.001$; n.s., difference not statistically significant.

Supplementary Table 1 – ParD is secreted specifically, but to a low extent

Label-free quantification (LFQ) data of proteins in the culture supernatant of wild-type *Y. enterocolitica* at the indicated time points after induction of T3SS expression by temperature shift to 37°C under the indicated conditions. For absolute abundance estimation, iBAQ values were extracted from the LFQ data set. ParD is compared to selected known T3SS effector proteins (top) and ribosomal proteins (controlling for the effect of cell lysis, bottom). Shades of blue indicate relative export levels for each protein. *n*=1.

Protein	secreting			non-secreting
	15 min	30 min	60 min	60 min
T3SS effector YopE	535293	1232408	4792588	41254
T3SS effector YopH	287279	647470	1727615	9927
T3SS effector YopQ	27146	89566	364073	1649
T3SS effector YopT	184	737	1212	76
T3SS-linked antitoxin ParD	0	8	189	5
50S ribosomal protein L21	2767	2783	1831	1756
50S ribosomal protein L24	9343	9659	8694	8574
50S ribosomal protein L2	5944	5853	4811	4782

Supplementary Table 2 – Cellular levels and ratio of ParD and ParE are not increased under secreting condition

Label-free quantification (LFQ) data obtained by shotgun proteomics-based mass spectrometry for cellular proteins expressed from the virulence plasmid in *Y. enterocolitica* ΔHOPEMTasd under non-secreting and secreting conditions, three hours after induction of T3SS expression by temperature shift to 37°C. Whereas most T3SS components are significantly upregulated under secreting conditions (Kudryashev *et al*, 2015), the cellular levels of ParD and ParE are not. Protein levels are expressed as protein intensities on a $\log_2$ scale. Proteins ordered according to their ratio of cellular levels under secreting and non-secreting conditions; individual replicate intensity values (repl.) are indicated.

Protein	Log_2 ratio intensity (secreting/ non-secreting)	Log_2 protein intensity non-secreting			Log_2 protein intensity secreting			$-Log_{10}$ *p* value
		repl. 1	repl. 2	repl. 3	repl. 1	repl. 2	repl. 3	
Translocator SctA (LcrV)	1.799	30.899	30.988	30.735	32.773	32.610	32.635	5.59
Translocator SctB (YopD)	1.334	30.827	30.709	30.681	32.055	32.114	32.049	5.70
T3SS component SctU	1.254	26.750	26.624	26.783	27.957	28.133	27.828	4.54
T3SS component SctN	1.206	28.320	28.227	28.265	29.395	29.520	29.514	5.47
T3SS component SctT	0.917	23.609	24.412	23.405	25.179	24.460	24.537	1.45
T3SS component SctK	0.905	28.660	28.679	28.372	29.507	29.400	29.519	3.71
T3SS component SctQ	0.833	31.150	31.031	30.981	31.953	31.865	31.844	4.38
T3SS component SctL	0.831	29.002	29.012	28.880	29.759	29.813	29.815	4.65
Exported regulator YscX	0.813	29.000	28.956	28.997	29.810	29.809	29.772	5.08
T3SS component SctQ	0.759	29.532	29.469	29.616	30.288	30.311	30.295	4.51
T3SS component SctJ	0.743	30.793	30.736	30.681	31.405	31.519	31.514	4.34
T3SS component SctD	0.715	28.281	28.309	28.134	29.013	28.807	29.047	3.41
Needle length regulator SctP	0.673	29.030	29.210	29.123	29.728	29.823	29.830	3.86
T3SS component SctR	0.660	26.290	26.143	26.467	27.008	26.947	26.926	3.17
T3SS component SctV	0.607	29.419	29.307	29.380	30.187	29.845	29.896	2.76
Needle subunit SctF	0.431	26.113	26.120	25.965	26.837	26.454	26.198	1.31
Pilotin protein SctG (YscW)	0.195	28.520	28.667	28.660	28.712	28.806	28.913	1.26
**T3SS-associated antitoxin ParD**	**0.046**	**28.816**	**28.776**	**28.888**	**28.798**	**28.936**	**28.885**	**0.29**
Adhesin YadA	-0.039	32.608	32.408	32.467	32.445	32.466	32.454	0.22
Gatekeeper SctW (YopN)	-0.252	28.705	28.997	28.725	28.739	28.542	28.390	1.00
**T3SS-associated toxin ParE**	**-0.297**	**29.029**	**29.135**	**29.115**	**28.850**	**28.751**	**28.788**	**2.45**
Negative regulator YscM2	-3.361	29.010	29.077	28.974	26.060	25.390	25.529	5.41
Negative regulator YscM1	-3.731	29.977	30.229	30.159	26.301	26.663	26.208	6.23

Supplementary Table 3 – Cellular levels and ratio of ParD and ParE are not affected by absence or overexpression of VirF, the main transcriptional regulator of the T3SS

Label-free quantification (LFQ) data obtained by shotgun proteomics-based mass spectrometry for cellular proteins expressed from the virulence plasmid in *Y. enterocolitica* ΔHOPEMTasd (WT), as well as bacteria lacking or overexpressing the main transcriptional activator of the T3SS, VirF (Δ*virF* and $VirF^+$, respectively), under secreting conditions, three hours after induction of T3SS expression by temperature shift to 37°C. Whereas most T3SS components are significantly downregulated in the absence of VirF, the cellular levels of ParD and ParE stay constant, similar to the levels of other non-T3SS-related proteins on the virulence plasmid. Protein levels are expressed as protein intensities on a $\log_2$ scale and ordered according to their ratio of cellular levels in presence and absence of VirF; individual replicate intensity values (repl.) are indicated.

Protein	Log_2 ratio intensity (WT/Δ*virF*)	Log_2 ratio intensity ($virF^+$/WT)	Log_2 protein intensity WT			Log_2 protein intensity Δ*virF*			Log_2 protein intensity $VirF^+$		
			repl. 1	repl. 2	repl. 3	repl. 1	repl. 2	repl. 3	repl. 1	repl. 2	repl. 3
T3SS component SctN	9.007	0.362	31.078	30.963	31.187	21.942	20.600	22.860	31.479	31.436	31.408
**Transcript. activator VirF**	**6.985**	**3.542**	**27.086**	**27.097**	**26.934**	**n.d.**	**19.620**	**n.d.**	**30.502**	**30.714**	**30.524**
T3SS component YscX	5.165	0.676	29.314	28.737	29.081	24.211	23.881	23.521	29.892	29.778	29.522
Translocator SctA (LcrV)	5.056	0.907	32.104	32.131	32.211	27.147	27.100	27.032	33.067	33.033	33.070
T3SS component SctV	4.967	0.208	30.974	30.686	30.849	26.238	25.886	25.368	31.087	31.038	31.019
T3SS component SctI	4.901	1.395	28.747	28.131	28.461	23.821	23.575	23.250	30.009	29.806	29.763
T3SS component SctD	4.746	0.225	30.008	29.730	29.879	25.190	25.355	24.792	30.091	30.100	30.115
Needle length regul. SctP	4.487	0.226	30.063	30.189	30.098	25.707	25.537	25.644	30.364	30.328	30.338
T3SS component SctC	4.475	0.182	31.675	31.228	31.419	27.444	26.738	26.604	31.635	31.613	31.655
T3SS component SctL	4.266	0.317	29.526	29.085	29.295	25.135	25.153	24.832	29.715	29.623	29.547
T3SS component SctK	4.243	0.352	26.878	26.554	26.829	22.217	22.701	22.590	27.161	27.144	27.029
T3SS component SctQ	3.917	0.017	29.141	29.046	28.928	24.811	25.030	25.456	28.919	29.090	29.154
Needle subunit SctF	3.784	0.084	30.661	30.460	30.492	27.609	26.058	25.963	30.530	30.629	30.706
T3SS component SctJ	3.349	0.291	31.319	30.897	31.137	28.031	27.760	27.496	31.466	31.382	31.407
T3SS component SctU	3.338	0.110	27.564	27.245	27.447	24.315	24.023	23.889	27.571	27.530	27.504
Resolvase TnpR	0.735	0.937	25.669	25.724	25.675	24.066	24.663	25.671	26.615	26.650	26.616
**T3SS-assoc. toxin ParE**	**0.352**	**-0.146**	**24.607**	**25.333**	**25.201**	**24.264**	**25.079**	**24.729**	**24.979**	**25.119**	**24.666**
**T3SS-assoc. antitoxin ParD**	**0.289**	**0.061**	**28.955**	**28.688**	**28.701**	**28.759**	**28.383**	**28.310**	**28.944**	**28.820**	**28.774**
Arsenic resist. prot. ArsH	0.274	0.277	26.329	26.340	26.203	26.053	25.925	26.070	26.578	26.583	26.544
Plasmid partit. prot. SpyA	0.141	0.085	28.709	28.588	28.611	28.529	28.481	28.479	28.743	28.712	28.711
Plasmid partit. prot. SpyB	0.126	-0.081	29.242	29.003	28.989	29.132	28.835	28.887	29.031	28.946	29.027
Arsenate reductase ArsC	-0.091	-0.200	27.517	27.505	27.423	27.714	27.474	27.520	26.781	27.513	27.447
Negative regulator YscM1	-0.534	0.631	28.225	27.871	27.444	28.460	28.301	28.480	28.742	28.503	28.251
Negative regulator YscM2	-1.218	1.675	25.476	25.783	25.708	26.659	26.953	27.004	27.600	27.277	27.085

Supplementary Table 4 – Effect of ParE overexpression on *Y. enterocolitica* proteome

Comparison of expression levels in total cellular protein samples of wild-type (WT) and additionally ParE-expressing ($ParE^+$) *Y. enterocolitica* under non-secreting conditions, determined by LFQ mass spectrometry.

All proteins with $\log_{10}(p)$>2.5 and a >2-fold change in cellular protein levels that were detected in all $ParE^+$ replicates (repl.) samples are included in the list, and ordered according to their expression ratio $ParE^+$/WT. Selected protein shaded according to cellular function: SOS-response (orange), T3SS-associated effectors or components (purple), ParDE TA system and other gyrase-associated protein (grey).

Protein	Log_2 ratio intensity ($ParE^+$/WT)	Log_2 protein intensity WT			Log_2 protein intensity $ParE^+$			$-Log_{10}$ *p* value
		repl. 1	repl. 2	repl. 3	repl. 1	repl. 2	repl. 3	
YEW_AN02710	6.328	23.305	24.269	24.224	30.207	30.582	29.994	5.65
L-arabinose uptake protein AraF	6.312	22.562	20.889	22.649	28.510	28.678	27.846	4.36
YEW_LE47450	6.299	23.779	23.647	23.613	29.893	29.954	30.088	8.66
**T3SS-associated toxin ParE**	**5.764**	**28.466**	**28.354**	**28.390**	**34.167**	**34.198**	**34.136**	**9.11**
DNA repair protein RecN	5.750	22.130	23.969	22.013	28.250	28.871	28.243	4.01
L-arabinose uptake protein AraB	5.731	23.174	23.637	22.947	28.954	29.134	28.865	6.52
YEW_FI22730	5.398	25.698	24.580	24.557	30.334	30.360	30.333	5.16
L-arabinose uptake protein AraD	4.955	23.575	23.996	24.028	28.620	28.975	28.869	6.58
T3SS-associated effector YopP	4.470	22.590	21.697	21.098	26.024	25.974	26.795	4.04
**T3SS-associated antitoxin ParD**	**4.399**	**28.822**	**28.953**	**28.833**	**33.242**	**33.241**	**33.323**	**8.27**
DNA-damage inducible protein DinI	4.228	25.232	24.425	24.957	29.320	29.129	28.849	5.30
Leucine transporter LivG	3.949	29.260	28.500	29.409	32.102	32.167	34.749	2.51
YEW_AN02690	3.927	24.045	24.477	24.248	28.482	28.173	27.897	5.70
YEW_JR41840	3.433	23.168	23.593	23.022	26.797	26.820	26.464	5.43
DNA polymerase DinB	3.269	23.131	23.178	22.867	26.026	26.018	26.940	4.35
Altronate oxidoreductase UxaB	3.148	24.205	22.465	23.521	26.808	26.881	25.945	2.95
Lysine decarboxylase ldcC	3.141	24.109	22.670	23.705	26.581	26.461	26.865	3.54
Molybdenum enzyme MobA	3.127	22.459	22.502	23.794	25.906	26.389	25.840	3.41
Acid response protein YhcN	2.872	25.999	26.162	25.933	28.830	28.594	29.287	4.93
YEW_LE47440	2.770	24.093	23.716	23.327	26.416	26.534	26.495	4.75
Arginine:ornithine antiporter AcrD	2.736	22.687	22.943	23.256	26.687	25.706	24.700	2.63

YEW_FU24450	2.598	23.515	23.547	23.741	27.280	26.099	25.220	2.51
DNA gyrase inhibitor SbmC	2.497	26.616	26.569	26.726	29.170	29.022	29.208	6.52
DNA-damage inducible protein YebG	2.486	24.263	23.838	24.162	26.263	26.740	26.718	4.73
YEW_AJ01320	2.466	24.632	23.055	23.474	25.983	26.146	26.432	2.82
Recombinase XerC	2.189	23.163	23.967	24.337	25.755	26.036	26.243	3.12
YEW_FU24460	2.111	24.095	24.508	24.060	26.535	26.064	26.399	4.35
DNA recombination protein RmuC	2.007	26.393	26.492	26.401	28.670	28.147	28.491	4.74
YEW_HU34330	1.990	28.504	28.338	28.243	30.313	30.275	30.468	5.59
Cell division protein FtsN homolog MsgA	1.948	23.474	23.637	24.621	25.913	25.689	25.973	2.91
Alcohol dehydrogenase YqhD	1.794	28.202	28.351	28.469	29.949	30.183	30.272	4.93
YEW_GE26310	1.747	27.838	27.062	26.980	29.199	28.640	29.282	2.84
DNA repair protein RecA	1.679	30.704	30.613	30.654	32.380	32.434	32.194	5.51
Peptide transport system OppC	1.413	23.807	22.938	23.619	24.773	24.895	24.933	2.87
Methionine sulfoxide reductase MsrA	1.359	24.315	24.690	24.545	25.804	25.841	25.982	4.31
Helicase UvrD involved in DNA repair	1.296	26.629	26.542	26.574	28.101	27.708	27.825	4.24
Galactokinase GalK	1.174	25.584	25.401	25.327	26.513	26.611	26.708	4.38
DNA damage sensor UvrB	1.171	27.763	27.702	27.755	29.028	28.755	28.951	4.57
Holiday junction helicase RuvA	1.120	25.108	25.299	25.463	26.398	26.477	26.355	4.06
YEW_FI22740	1.100	23.411	23.224	23.601	24.400	24.454	24.683	3.59
YEW_EW21010	1.100	28.879	28.781	28.388	29.724	29.746	29.878	3.36
Acetyltransferase YhhY	1.094	25.734	24.993	25.648	26.465	26.645	26.547	2.55
YEW_FE21930	1.063	26.577	26.799	26.232	27.846	27.273	27.677	2.52
L-threonine dehydratase TdcB	1.047	24.668	25.333	25.308	26.064	26.201	26.184	2.61
UDP-galactose-4-epimerase GalE	1.045	26.060	25.886	26.052	27.023	27.158	26.953	4.32
T3SS-associated effector YopQ	1.043	28.462	28.324	28.510	29.344	29.470	29.610	4.11
DNA damage sensor UvrA	1.016	28.507	28.335	28.384	29.829	29.225	29.219	2.66
YEW_AY04760	-1.016	27.361	27.662	27.644	26.771	26.624	26.225	2.84
YEW_IJ36440	-1.020	27.017	26.827	26.787	25.775	25.717	26.078	3.51
YEW_HJ32820	-1.023	28.761	28.887	28.777	27.851	27.616	27.887	4.08
YEW_GS28730	-1.091	28.138	28.197	28.161	27.152	27.270	26.802	3.54

Gatekeeper protein YopN	-1.098	30.512	30.649	30.618	29.377	29.729	29.379	3.79
Urease accessory protein UreE	-1.106	30.102	30.046	30.043	28.767	29.078	29.030	4.18
acetyl-CoA C-acyltransferase FadI	-1.135	25.030	25.266	24.975	23.972	23.983	23.913	4.35
Urease accessory protein UreF	-1.137	28.004	28.118	28.122	26.795	27.155	26.884	4.00
E3 ubiquitin-protein ligase Cbl	-1.203	25.912	25.586	25.604	24.251	24.429	24.814	3.13
Pyruvate-inducible protein YhjX	-1.216	25.516	25.333	25.515	24.502	24.148	24.065	3.71
Fe-S cluster assembly regulator IscR	-1.238	28.017	28.067	28.137	26.647	27.018	26.842	4.23
YEW_AY04770	-1.239	28.606	28.050	27.873	26.653	27.237	26.923	2.53
Urease accessory protein UreD	-1.240	28.140	28.073	28.334	26.909	27.027	26.890	4.60
Stationary phase associated YqjD	-1.281	33.308	34.174	33.946	32.333	32.665	32.586	2.60
YEW_GL27900	-1.292	27.320	27.688	27.936	26.080	26.193	26.797	2.56
YahO	-1.301	29.265	29.554	29.685	28.172	28.287	28.144	4.07
Bacterioferritin Bfr	-1.312	28.657	28.489	28.583	27.405	27.083	27.305	4.45
Alpha-amylase AmyA	-1.746	25.451	25.386	25.978	23.286	24.064	24.229	2.80
Ornithine decarboxylase SpeF	-1.945	25.524	25.372	25.182	23.718	22.844	23.681	3.31
YEW_CY12230	-2.112	27.718	27.702	27.190	25.650	25.536	25.087	3.95
Repressor of SOS-regulated-genes LexA	-2.468	27.735	27.746	27.621	25.076	25.221	25.401	6.00

Supplementary Table 5 – Strains and plasmids used in this study

Plasmid	Genotype and characteristics	Reference
pBAD	Cloning and expression vector for *Yersinia*, Ampicillin resistance, arabinose inducible	Invitrogen
pAD003	*pKNG101-ΔvirF* (mutator for deletion of the *virF* gene)	This study
pAD633	*pBAD::YopO*$_{1\text{-}19}$*-FLAG-YopO*$_{78\text{-}728}$*-SctP*$_{308\text{-}381}$ (control vector for needle plugging)	This study
pAD642	*pBAD::YopO*$_{1\text{-}19}$*-FLAG-YopO*$_{78\text{-}728}$*-SctP*$_{308\text{-}381}$*-EGFP* (vector for needle plugging)	This study
pAD700	*pKNG101-ΔparE* (mutator for deletion of the *parE* gene)	This study
pAD702	*pKNG101-ΔparDE* (mutator for deletion of the *parD* and *parE* gene)	This study
pAD709	*pBAD::parE* (vector for ParE overexpression, arabinose inducible promotor)	This study
pAD719	*pBAD::parD* (vector for ParD overexpression, arabinose inducible promotor)	This study
pAD720	*pBAD::parD-FLAG* (vector for ParD-FLAG overexpression, arabinose inducible promotor)	This study
pAD725	*pBAD::sycE-yopE*$_{1\text{-}138}$*-HiBiT* (vector for HiBiT luciferase complementation)	This study
pFE010	*pBAD::virF* (vector for VirF overexpression, arabinose inducible promoter)	This study

Strain	Genotype	Reference
MRS40	Wild-type *Y. enterocolitica* E40 (pYVe40) Δ*blaA*	Sory *et al*, 1995
IML421*asd* (ΔHOPEMTasd)	pYVmrs40 *yopO*$_{\Delta 2\text{–}427}$ *yopE*$_{21}$ *yopH*$_{\Delta 1\text{–}352}$ *yopM*$_{23}$ *yopP*$_{23}$ *yopT*$_{135}$ *Δasd*	Kudryashev *et al*, 2013
AD4051	pYVmrs40 *ΔsctD*	Diepold *et al*, 2010
AD4085	pYViml421asd *egfp-sctQ*	Kudryashev *et al*, 2013
AD4618	pYViml421asd *egfp-sctQ* Δ*parE*	This study
AD4619	pYViml421asd *egfp-sctQ* Δ*parDE*	This study
FE014	pYViml421asd *ΔvirF*	This study
pIM41	pYVmrs40 *ΔsctW (yopN)*	Boland *et al*, 1996

Supplementary information references

Boland A, Sory M-P, Iriarte M, Kerbourch C, Wattiau P & Cornelis GR (1996) Status of YopM and YopN in the Yersinia Yop virulon: YopM of Y.enterocolitica is internalized inside the cytosol of PU5-1.8 macrophages by the YopB, D, N delivery apparatus. *EMBO J.* **15:** 5191–201

Diepold A, Amstutz M, Abel S, Sorg I, Jenal U & Cornelis GR (2010) Deciphering the assembly of the Yersinia type III secretion injectisome. *EMBO J.* **29:** 1928–40

Diepold A, Sezgin E, Huseyin M, Mortimer T, Eggeling C & Armitage JP (2017) A dynamic and adaptive network of cytosolic interactions governs protein export by the T3SS injectisome. *Nat. Commun.* **8:** 15940

Kudryashev M, Diepold A, Amstutz M, Armitage JP, Stahlberg H & Cornelis GR (2015) Yersinia enterocolitica type III secretion injectisomes form regularly spaced clusters, which incorporate new machines upon activation. *Mol. Microbiol.* **95:** 875–884

Kudryashev M, Stenta M, Schmelz S, Amstutz M, Wiesand U, Castaño-Díez D, Degiacomi MT, Münnich S, Bleck CK, Kowal J, Diepold A, Heinz DW, Dal Peraro M, Cornelis GR & Stahlberg H (2013) In situ structural analysis of the Yersinia enterocolitica injectisome. *Elife* **2:** e00792

Sory M-P, Boland A, Lambermont I & Cornelis GR (1995) Identification of the YopE and YopH domains required for secretion and internalization into the cytosol of macrophages, using the cyaA gene fusion approach. *Proc. Natl. Acad. Sci. U. S. A.* **92:** 11998–12002
